# Characterization of Emetic and Diarrheal *Bacillus cereus* Strains From a 2016 Foodborne Outbreak Using Whole-Genome Sequencing: Addressing the Microbiological, Epidemiological, and Bioinformatic Challenges

**DOI:** 10.3389/fmicb.2019.00144

**Published:** 2019-02-12

**Authors:** Laura M. Carroll, Martin Wiedmann, Manjari Mukherjee, David C. Nicholas, Lisa A. Mingle, Nellie B. Dumas, Jocelyn A. Cole, Jasna Kovac

**Affiliations:** ^1^Department of Food Science, Cornell University, Ithaca, NY, United States; ^2^Department of Food Science, The Pennsylvania State University, University Park, PA, United States; ^3^New York State Department of Health, Corning Tower, Empire State Plaza, Albany, NY, United States; ^4^New York State Department of Health, Wadsworth Center, Albany, NY, United States

**Keywords:** *Bacillus cereus*, foodborne outbreak, whole-genome sequencing, emetic disease, cytotoxicity, SNP calling, genomic epidemiology

## Abstract

The Bacillus cereus group comprises multiple species capable of causing emetic or diarrheal foodborne illness. Despite being responsible for tens of thousands of illnesses each year in the U.S. alone, whole-genome sequencing (WGS) is not yet routinely employed to characterize *B. cereus* group isolates from foodborne outbreaks. Here, we describe the first WGS-based characterization of isolates linked to an outbreak caused by members of the *B. cereus* group. In conjunction with a 2016 outbreak traced to a supplier of refried beans served by a fast food restaurant chain in upstate New York, a total of 33 *B. cereus* group isolates were obtained from human cases (*n* = 7) and food samples (*n* = 26). Emetic (*n* = 30) and diarrheal (*n* = 3) isolates were most closely related to *B. paranthracis* (group III) and *B. cereus sensu stricto* (group IV), respectively. WGS indicated that the 30 emetic isolates (24 and 6 from food and humans, respectively) were closely related and formed a well-supported clade distinct from publicly available emetic group III genomes with an identical sequence type (ST 26). The 30 emetic group III isolates from this outbreak differed from each other by a mean of 8.3 to 11.9 core single nucleotide polymorphisms (SNPs), while differing from publicly available emetic group III ST 26 *B. cereus* group genomes by a mean of 301.7–528.0 core SNPs, depending on the SNP calling methodology used. Using a WST-1 cell proliferation assay, the strains isolated from this outbreak had only mild detrimental effects on HeLa cell metabolic activity compared to reference diarrheal strain *B. cereus* ATCC 14579. We hypothesize that the outbreak was a single source outbreak caused by emetic group III *B. cereus* belonging to the *B. paranthracis* species, although food samples were not tested for presence of the emetic toxin cereulide. In addition to showcasing how WGS can be used to characterize *B. cereus* group strains linked to a foodborne outbreak, we also discuss potential microbiological and epidemiological challenges presented by *B. cereus* group outbreaks, and we offer recommendations for analyzing WGS data from the isolates associated with them.

## Introduction

The *Bacillus cereus* (*B. cereus*) group, also known as *B. cereus sensu lato* (*s.l*.) is a complex of closely related species that vary in their ability to cause disease in humans. Foodborne illness caused by members of the group primarily manifests itself in one of two forms: (i) emetic intoxication that is caused by cereulide, a heat-stable toxin produced by *B. cereus* within a food matrix prior to consumption, or (ii) a diarrheal toxicoinfection, caused by enterotoxins produced by bacteria in the small intestine of the host (Ehling-Schulz et al., 2004; Schoeni and Wong, 2005; Stenfors Arnesen et al., 2008). Here we refer to isolates that carry *ces* genes encoding the cereulide biosynthetic pathway as emetic isolates, and isolates that lack *ces* genes but carry either *hbl* or *cytK-2* genes that encode diarrheal enterotoxins as diarrheal isolates. The gene variant *cytK-2* was included in this definition, as it was previously found in non-*B. cytotoxicus* isolates associated with diarrheal illness (Castiaux et al., 2015; Miller et al., 2018). The presence of *nhe* genes was not included in our present definition of diarrheal isolates, due to the fact that *nhe* genes are ubiquitously found in the majority of the *B. cereus* group population (Carroll et al., 2017; Miller et al., 2018), including all isolates in the present study, and their contribution to diarrheal toxicoinfection is not yet fully understood (Doll et al., 2013).

As foodborne pathogens, members of the *B. cereus* group are estimated to cause 63,400 foodborne disease cases per year in the U.S. (Scallan et al., 2011) and are confirmed or suspected to have been responsible for 235 outbreaks reported in the U.S. between 1998 and 2008 (Bennett et al., 2013). Due in part to its typically self-limiting nature, foodborne illness caused by members of the *B. cereus* group is under-reported (Granum and Lund, 1997; Stenfors Arnesen et al., 2008), although severe infections resulting in patient death have been reported (Naranjo et al., 2011; Sanaei-Zadeh, 2012; Lotte et al., 2017). Furthermore, *B. cereus* group isolates that have been linked to human clinical cases of foodborne disease rarely undergo whole-genome sequencing (WGS), as is becoming the norm for other foodborne pathogens (Joensen et al., 2014; Ashton et al., 2015; Moura et al., 2017).

Here, we describe a foodborne outbreak caused by members of the *B. cereus* group in which WGS was implemented to characterize isolates from human clinical cases and food. To our knowledge, this is the first description of a *B. cereus* outbreak in which WGS was employed to characterize isolates. By testing various combinations of variant calling methodologies, we showcase how different bioinformatics pipelines can yield vastly different results when pairwise SNP differences are the desired metric for determining whether an isolate is part of an outbreak or not. In addition to discussing the bioinformatic challenges, we examine potential microbiological and epidemiological obstacles that can hinder characterization of *B. cereus* group isolates from suspected foodborne outbreaks, and we offer recommendations to guide the characterization of future *B. cereus* group outbreaks using WGS.

## Materials and Methods

### Collection of Epidemiological Data

Epidemiological investigations were coordinated by the New York State Department of Health (NYSDOH), and the outbreak was reported to the U.S. Centers for Disease Control and Prevention (CDC). Investigation methods included (i) a cohort study, (ii) food preparation review, (iii) an investigation at a factory/production/treatment plant, (iv) food product traceback, and (v) environment/food/water sample testing.

### Isolation and Initial Characterization of *B. cereus* Strains

Stool specimens were plated directly onto mannitol-egg yolk-polymyxin (MYP) agar and incubated aerobically at 37°C for 24 h. Food samples were diluted 1:10 in 1 X PBS, pH 7.4 in a filter bag for homogenizer blenders and homogenized for 2 min. One hundred μl of each homogenized sample were plated onto MYP agar and incubated aerobically at 37°C for 24 h. The MYP agar plates for both the stool specimens and food samples were observed after the 24 h incubation period. Individual *B. cereus*-like colonies (i.e., pink colored and lecithinase positive) were subcultured on trypticase soy agar (TSA) plates supplemented with 5% sheep blood and incubated aerobically at 37°C for 18–24 h. These isolates were identified as *B. cereus* using the following conventional microbiological techniques: Gram stain, colony morphology, hemolysis, motility, and spore stain. To test for the presence of parasporal crystals often associated with *B. thuringiensis*, isolates were cultured for 48 h at 37°C on sporulation agar slants. Smears were prepared, and slides were heat fixed and then stained using malachite green and counter stained with carbol fuchsin (Tallent et al., 2012b). Slides were then observed for the presence or absence of parasporal crystals.

### *rpoB* Allelic Typing

The 33 outbreak isolates were streaked onto brain heart infusion (BHI) agar from their respective cryo stocks stored at −80°C and incubated overnight at 37°C. Single isolated colonies were inoculated in 5 ml BHI broth and incubated overnight at 32°C and used for genomic DNA extraction using Qiagen DNeasy blood and tissue kits (Qiagen). Extracted DNA was used as a template in a PCR reaction using primers targeting a 750 bp sequence of the *rpoB* gene (RzrpoBF: AARYTIGGMCCTGAAGAAAT and RZrpoBR: TGIARTTRTCATCAACCATGTG) (Ivy et al., 2012). PCR was carried out in 25 μl reactions using GoTaq Green Master Mix (Promega Corporation) under the following thermal cycling conditions: 3 min at 94°C, followed by 40 cycles of 30 s at 94°C, 30 s at 55–45°C (in the first 20 cycles the temperature was reduced for 0.5°C per cycle and then kept at 45°C in the following 20 cycles), followed by 1 min at 72°C, and a final hold at 4°C. The resulting PCR product was used for genotyping and preliminary species identification using the *rpoB* allele type database available in Food Microbe Tracker (Ivy et al., 2012; Vangay et al., 2013).

### Bacterial Growth Conditions and Collection of Bacterial Supernatants

The 33 outbreak isolates, as well as *B. cereus* s.s. type strain ATCC 14579 and *B. cereus* emetic reference strain DSM 4312 (Food Microbe Tracker ID FSL M8-0547; Vangay et al., 2013) were streaked onto BHI agar from their respective cryo stocks stored at −80°C. For immunoassays and cytotoxicity assays (see sections Hemolysin BL and Non-hemolytic Enterotoxin Detection and WST-1 Metabolic Activity Assay), cultures grown from single isolated colonies for 18 h at 37°C without shaking were used for inoculation of fresh BHI broth. Fresh cultures were grown to early stationary phase as determined by an OD600 of ~1.5, which equals ~10^8^ CFU/ml. After incubation, growth was quenched by placing cultures on ice. The cultures were then spun down at 16,000 g for 2 min, and the supernatants were collected, aliquoted in duplicate, and stored at −80°C until further use in cytotoxicity assays.

### Hemolysin BL and Non-hemolytic Enterotoxin Detection

Diarrheal strains grown as described above were used for qualitative detection of hemolysin BL (Hbl) and non-hemolytic enterotoxins (Nhe) with the Duopath Cereus Enterotoxins immunoassay (Merck). Only select representatives of emetic outbreak strains were tested (i.e., FSL R9-6381, FSL R9-6382, FSL R9-6384, FSL R9-6389, FSL R9-6395, and FSL R9-6399), as they did not carry genes encoding Hbl and were therefore not expected to produce Hbl. Briefly, the temperatures of the cultures and immunoassay kits were adjusted to room temperature. 150 μl of each isolate culture were added to the immunoassay port, following the manufacturer's instructions. The results were read as positive if a red test line was visible after a 30-min incubation at room temperature. Tests were considered valid only when control lines were visible.

### WST-1 Metabolic Activity Assay

HeLa cells were seeded in 96-well plates at a seeding density of 8 × 10^4^ cells/cm^2^ (Fisichella et al., 2009) in Eagle's minimum essential medium (EMEM) supplemented with 10% fetal bovine serum (FBS) and allowed to grow for 18–24 h at 37°C, 5% CO_2_. After incubation, the medium in each well was replaced with 100 μl of fresh medium containing 5% v/v of bacterial supernatants (prepared as described in section Bacterial Growth Conditions and Collection of Bacterial Supernatants) that were thawed and pre-warmed to 37°C. The combined medium and supernatants were added to the cells using a multichannel pipettor to minimize the variability in the duration of cell exposure to the toxin amongst wells of a 96-well plate. Medium containing 5% BHI was used as a negative control. Medium containing 5% v/v of 1% Triton X-100 dissolved in BHI (final concentration in the test well was 0.05%) was used as a positive control expected to significantly reduce the viability of HeLa cells. After 15 min of intoxication at 37°C, 5% CO_2_ (Miller et al., 2018), 10 μl of WST-1 dye solution (Roche) was added to each well of the plate, and the plate was incubated for 25 min at 37°C, 5% CO_2_, resulting in a total of 40 min exposure of cells to the supernatants. After 30 s of orbital shaking at 600 rpm, the absorbances were read by a microplate reader (Thermo Scientific Multiskan GO, Thermo Fisher Scientific) in a precision mode at 450 and 690 nm, the latter being subtracted from the former to account for the background signal (i.e., corrected absorbances) (Fisichella et al., 2009). Each test, including 0.05% Triton X-100, was conducted with six technical replicates and on two different HeLa passages using supernatants from single biological replicates, resulting in a total of 12 technical replicates per isolate. The viability of cells was determined by calculating a ratio of corrected absorbances to that of BHI, converting to percentages, and calculating the mean of the technical replicates for each isolate. The results were compared to the results for cells treated with (i) 0.05% Triton X-100, (ii) *B. cereus* s.s. type strain ATCC 14579 supernatant (i.e., reference for diarrheal strains), and (iii) *B. cereus* group strain DSM 4312 supernatant (i.e., reference for emetic strains).

### Statistical Analysis of Cytotoxicity Data

A Welch's test and the Games-Howell *post-hoc* test that are appropriate for analyses of data with non-homogeneous variances were performed using results of all 12 technical replicates of each outbreak-associated isolate, as well as the reference strains and the positive control. For the Games-Howell test, a Bonferroni correction was applied to correct for multiple comparisons. Statistical analyses were carried out in R version 3.4.3 (R Core Team., 2018).

### Whole-Genome Sequencing

Genomic DNA was extracted from overnight cultures (~18 h) grown in BHI at 32°C using Qiagen DNeasy blood and tissue kits (Qiagen) or the Omega E.Z.N.A. Bacterial DNA kit (Omega) following the manufacturers' instructions. For the E.Z.N.A. Bacterial DNA kit, the additional steps recommended for difficult-to-lyse bacteria were taken to obtain sufficient DNA yield. Briefly, one ml of an overnight culture was additionally treated with glass beads provided in the E.Z.N.A. kit. DNA was quantified using Qubit 3 and used for Nextera XT library preparation (Illumina). Pooled libraries were sequenced in two Illumina sequencing runs with either 2 × 250 or 2 × 300 bp reads at the Penn State Genomics Core Facility and at the Cornell Animal Health Diagnostic Center.

### Initial Data Processing and Genome Assembly

Illumina adapters and low-quality bases were trimmed using Trimmomatic version 0.36 (Bolger et al., 2014) and the default parameters for Nextera paired-end reads, and FastQC version 0.11.5 (https://www.bioinformatics.babraham.ac.uk/projects/fastqc/) was used to confirm that read quality was adequate (e.g., no reads flagged as poor quality, no Illumina adapters present). Genomes listed in Supplementary Table S1 were assembled *de novo* using SPAdes version 3.11.0 (Bankevich et al., 2012), and average per-base coverage was calculated using Samtools version 1.6 (Li et al., 2009) after mapping reads to their respective *de novo* assemblies using BWA MEM version 0.7.13 (default parameters) (Li and Durbin, 2010).

### *In silico* Typing and Virulence Gene Detection

BTyper version 2.2.0 (Carroll et al., 2017) was used to perform *in silico* virulence gene detection, multi-locus sequence typing (MLST), *panC* group assignment (as defined by Guinebretiere et al., 2010), and *rpoB* allelic typing, as well as to extract the gene sequences for all detected loci. For virulence gene detection, the default settings were used (i.e., 50% amino acid sequence identity, 70% query coverage), as these cut-offs have been shown to correlate with PCR-based detection of virulence genes in *B. cereus* group isolates (Kovac et al., 2016; Carroll et al., 2017). BMiner version 2.0.2 (Carroll et al., 2017) was used to aggregate the output files from BTyper and create a virulence gene presence/absence matrix.

### Construction of *k*-mer Based Phylogeny Using Outbreak Strains and Genomes of 18 *B. cereus* Group Species

kSNP version 3.1 (Gardner and Hall, 2013; Gardner et al., 2015) was used to produce a set of core SNPs among the 33 outbreak genomes, plus a type strain or RefSeq reference genome assembly from each of the 18 *B. cereus* group species listed in Supplementary Table S2 (Stenfors Arnesen et al., 2008; Guinebretière et al., 2013; Jiménez et al., 2013; Miller et al., 2016; Liu et al., 2017), using the optimal k-mer size as determined by Kchooser (*k* = 21). The resulting core SNPs were used in conjunction with RAxML version 8.2.11 (Stamatakis, 2014) to construct a maximum likelihood (ML) phylogeny using the GTRCAT model with a Lewis ascertainment bias correction (Lewis, 2001) to account for the use of solely variant sites, and 500 bootstrap replicates. The resulting phylogenetic tree was formatted using the phylobase (Hackathon et al., 2017), ggtree (Guangchuang et al., 2017), phytools (Revell, 2012), and ape (Paradis et al., 2004) packages in R version 3.4.3.

### Variant Calling and Phylogeny Construction Using Outbreak Isolates

Combinations of five reference-based variant calling pipelines (Table 1) and reference genomes (Table 2), as well as one reference-free SNP calling pipeline (Table 1), were used to separately identify core and total SNPs among (i) all 33 outbreak-related isolates (30 emetic group III isolates and three group IV isolates) and (ii) the subset of 30 emetic group III isolates. For the subset of 30 emetic group III isolates, all reference-based variant calling pipelines described below were additionally run with dustmasked versions of the reference genomes listed in Table 2, in which DustMasker version 1.0.0 (part of BLAST version 2.6.0) (Morgulis et al., 2006) was used to mask low-complexity portions (i.e., intervals with highly biased nucleotide distributions which can bias sequence similarity searches) in each reference genome (Ye et al., 2006).

**Table 1:** Variant calling pipelines tested in this study.

**Pipeline^a^**	**Approach**	**Reference-based**	**Input data (file format)^b^**	**Read mapper**	**Variant caller**	**Reference(s) and in-depth pipeline descriptions**
CFSAN	Read mapping	Yes	PE reads (fastq)	Bowtie2	Varscan	http://snp-pipeline.readthedocs.io/en/latest/
Freebayes	Read mapping	Yes	PE reads (fastq)	BWA MEM	Freebayes	https://github.com/lmc297/SNPBac
kSNP3	*k-*mer based	No	Contigs (fasta)	Not applicable	kSNP3	https://sourceforge.net/projects/ksnp/files/
LYVE-SET	Read mapping	Yes	PE reads (fastq)	SMALT	Varscan	https://github.com/lskatz/lyve-SET
Parsnp	Core genome alignment	Yes	Contigs (fasta)	Not applicable	Parsnp	http://harvest.readthedocs.io/en/latest/content/parsnp.html
Samtools	Read mapping	Yes	PE reads (fastq)	BWA MEM	Samtools/Bcftools	https://github.com/lmc297/SNPBac

a *CFSAN, U.S. Food and Drug Administration (FDA) Center for Food Safety and Applied Nutrition SNP pipeline; LYVE-SET, U.S. Centers for Disease Control and Prevention (CDC) Listeria, Yersinia, Vibrio, and Enterobacteriaceae SNP Extraction Tool*.

b *PE reads, Illumina paired-end reads*.

**Table 2:** Reference genomes used for reference-based variant calling in this study.

**Reference genome**	**Phylogenetic group^a^**	**Data set(s)^b^**	**ANI range^c^**	**NCBI accession number**	**Assembly level**	**Rationale for selection**
*B. cereus* strain ATCC 14579 chromosome	IV	All 33 isolates from two groups (groups III and IV)	98.8–98.9 (group IV) 91.8–92.3 (group III)	NC_004722.1	Complete Genome	*B. cereus s.s*. type strain; RefSeq reference genome; member of *panC* group IV, the same group as the three non-emetic outbreak-associated isolates sequenced in this study
*B. cereus* strain AH187 chromosome	III	All 33 isolates from two groups (groups III and IV); 30 emetic group III isolates	92.0–92.2 (group IV) 99.8–99.9 (group III)	NC_011658.1	Complete Genome	Human clinical isolate associated with an emetic outbreak in 1972 (cooked rice, United Kingdom); identical virulotype, MLST sequence type, *rpoB* allelic type, and *panC* group as 30 emetic outbreak isolates sequenced in this study
*B. cytotoxicus* strain NVH 391-98 chromosome	VII	All 33 isolates from two groups (groups III and IV)	82.6–82.7 (group IV) 82.5–82.9 (group III)	NC_009674.1	Complete Genome	Type strain of *B. cytotoxicus*, the most distant member of the *B. cereus* group as currently defined; shares a common ancestor with all isolates sequenced in this study
FOOD_10_19_16_RSNT1_2H_R9-6393	III	30 emetic group III isolates	92.0–92.2 (group IV) 100^d^-100 (group III)	SRR6825038	Contigs	Emetic isolate from the outbreak reported here; assembly had high per-base coverage, as well as the fewest number of contigs of all genome assemblies from isolates in this outbreak

a Phylogenetic group determined via panC group assignment function in BTyper version 2.2.0.

b *Data set(s) in this study for which given genome was used as a reference genome for reference-based SNP calling*.

c *Minimum and maximum average nucleotide identity (ANI) values of reference strain relative to group IV and group III genomes sequenced in this outbreak (n = 3 and 30, respectively) calculated using FastANI*.

d *Minimum ANI value was <100 prior to rounding*.

For the Samtools and Freebayes pipelines (Table 1), trimmed Illumina paired-end reads from the queried isolates were mapped to the appropriate reference genome using BWA mem version 0.7.13 (Li, 2013) and either Samtools/Bcftools version 1.6 (Li et al., 2009) or Freebayes version 1.1.0 (Garrison and Marth, 2012), respectively, were used to call variants. Vcftools version 0.1.14 (Danecek et al., 2011) was used to remove indels and SNPs with a SNP quality score <20, as well as to construct consensus sequences. For both variant calling pipelines, Gubbins version 2.2.0 (Croucher et al., 2015) was used to remove recombination events from the consensus sequences, and the Neighbor Similarity Score (NSS) (Jakobsen and Easteal, 1996), Maximum Chi-Squared (Smith, 1992), and Pairwise Homoplasy Index (PHI) (Bruen et al., 2006) tests implemented in PhiPack version 1.0 (Bruen et al., 2006) were used to assess whether recombination and homoplasies were present in sequence alignments before and after recombination was removed, using 1,000 permutations each and a window size of 100 (Supplementary Table S3). Both of these pipelines are publicly available and can be reproduced in their entirety (SNPBac version 1.0.0; https://github.com/lmc297/SNPBac).

For the CFSAN (Davis et al., 2015) and LYVE-SET (Katz et al., 2017) pipelines (versions 1.0.1 and 1.1.4 g, respectively; Table 1), trimmed Illumina paired-end reads were used as input, and all default pipeline steps were run as outlined in the manuals. For the Parsnp pipeline (Treangen et al., 2014) (Table 1), assembled genomes of the outbreak isolates were used as input, and Parsnp's implementation of PhiPack (Bruen et al., 2006) was used to filter out recombination events. For kSNP3 (Table 1), assembled genomes of the outbreak isolates were used as input, and Kchooser was used to determine the optimum *k*-mer size for the full 33-isolate data set and the 30 emetic group III isolate set (*k* = 21 and 23, respectively).

For all variant calling and filtering pipelines, RAxML version 8.2.10 was used to construct ML phylogenies using the resulting SNPs under the GTRGAMMA model with a Lewis ascertainment bias correction and 1,000 bootstrap replicates. Phylogenetic trees were annotated using FigTree version 1.4.3 (http://tree.bio.ed.ac.uk/software/figtree/).

### Variant Calling and Statistical Comparison of Emetic Outbreak Isolates to Publicly Available Genomes

To compare emetic group III isolates from this outbreak to other emetic group III isolates, BTyper version 2.2.1 was used to query all 2,156 *B. cereus* group genome assemblies available in NCBI's RefSeq database (downloaded March 2018) (Pruitt et al., 2007) and identify all genome assemblies that (i) belonged to group III based on *panC* sequence, (ii) belonged to ST 26 based on *in silico* MLST, and (iii) were found to possess the *ces* operon in its entirety (*cesABCD*) at the default coverage and identity thresholds. This search produced 25 genome assemblies in addition to the 30 emetic group III genomes sequenced here. Only three of the 25 RefSeq genome assemblies had Sequence Read Archive (SRA) data linked to their BioSample accession numbers, making short read data readily available only for these three isolates. Consequently, only Parsnp version 1.2 and kSNP version 3.1 were used to identify SNPs in all 55 group III emetic genomes (25 from NCBI RefSeq and 30 sequenced here), as these approaches can be used with assembled genomes and do not require short reads as input. For Parsnp, the chromosome of *B. cereus* AH187 was used as a reference genome. For kSNP3, Kchooser was used to select the optimal k-mer size (*k* = 21), and the chromosome of *B. cereus* AH187 was included for *k*-mer based SNP calling.

RAxML version 8.2.10 was used to construct ML phylogenies using the resulting core SNPs for each of the Parsnp and kSNP3 pipelines under the GTRCAT model with a Lewis ascertainment bias correction and 1,000 bootstrap replicates. Pairwise core SNP differences between all 55 isolates were obtained using the dist.gene function in R's ape package. The permutest and betadisper functions in R's vegan package (Oksanen et al., 2018) were used to conduct an ANOVA-like permutation test to test if publicly available genomes were more variable than isolates from this outbreak based on pairwise core SNP differences and 5 independent trials using 100,000 permutations each. Analysis of similarity (ANOSIM; Clarke, 1993) using the anosim function in the vegan package in R was used to determine if the average of the ranks of within-group distances was greater than or equal to the average of the ranks of between-group distances (Anderson and Walsh, 2013), where groups were defined as (i) the 30 emetic isolates from this outbreak, and (ii) the 25 external emetic ST 26 isolates (downloaded from RefSeq). ANOSIM tests were conducted using pairwise core SNP differences and five independent runs of 10,000 permutations each. For both the ANOVA-like permutation tests and the ANOSIM tests, Bonferroni corrections were used to correct for multiple comparisons at the α = 0.05 level.

### Statistical Comparison of Phylogenetic Trees

The Kendall-Colijn (Kendall and Colijn, 2015) test described by Katz et al. (2017) was used to compare the topologies of trees, using the treespace (Jombart et al., 2017), ips (Heibl, 2008), phangorn (Schliep, 2011), docopt (de Jonge, 2016), and stringr (Wickham, 2018) packages in R version 3.4.3. The phylogenies that underwent pairwise testing were constructed using (i) either core or total SNPs identified in 30 emetic group III genomes via all six SNP calling pipelines (Table 1), using either an unmasked or dustmasked closed reference genome (*B. cereus* AH187; Table 2), and (ii) SNPs identified in 55 emetic ST 26 genomes (25 publicly available genomes and the 30 emetic isolates sequenced here) using the kSNP3 (core and total SNPs) and Parsnp (core SNPs, as Parsnp queries the core genome by definition) pipelines. For all pairwise tree comparisons, a lambda value of 0 (to give weight to tree topology rather than branch lengths; Katz et al., 2017) was used along with 100,000 random trees as a background distribution, and a Bonferroni correction was used to correct for multiple comparisons. Pairs of trees were considered to be more topologically similar than would be expected by chance if a significant *P*-value (*P* < 0.05) resulted after correcting for multiple testing (Katz et al., 2017).

### Calculation of Average Nucleotide Identity Values

FastANI version 1.0 (Jain et al., 2018) was used to calculate average nucleotide identity (ANI) values between assembled genomes of isolates sequenced in this study and selected reference genomes (Table 2), as well as the genomes of 18 currently published *B. cereus* group species (Supplementary Table S2).

### Availability of Data

Trimmed Illumina reads for all 33 isolates sequenced in this study have been made publicly available (NCBI BioProject Accession PRJNA437714), with NCBI BioSample and SRA accession numbers for all isolates listed in Supplementary Table S1. All figures have been deposited in FigShare (DOI https://doi.org/10.6084/m9.figshare.7001525.v1), and records of all isolates are available in Food Microbe Tracker (Vangay et al., 2013).

## Results

### Both Emetic and Diarrheal Symptoms Were Reported Among Cases Associated With the *B. cereus* Foodborne Outbreak

Between September 30th and October 6th, 2016, local health departments in upstate New York's Niagara and Erie counties reported a total of 179 estimated foodborne illness cases among customers of a Mexican fast-food restaurant chain in eight towns/cities. Among these cases, laboratory results were available for ten cases. For seven of these cases, *B. cereus* group species were isolated from patient stool samples. While no deaths, hospitalizations, or emergency room visits were reported from 169 cases from which information was obtained, 4 resulted in a visit to a health care provider (not including emergency room visits). More than 2/3 of 179 cases were female (69%), and 61% of cases fell within the 20–74 age group. In 156 of 179 total cases (87%), refried beans had been consumed.

Of 169 cases from which information was obtained, 88% reported vomiting, and more than half reported nausea and abdominal cramps (95 and 65%, respectively). However, in addition to vomiting, 38% of cases also reported diarrhea. Additional symptoms reported included (i) weakness (43%), (ii) chills (40%), (iii) dehydration (35%), (iv) headache (28%), (v) myalgia (muscle ache/pain; 16%), (vi) fever (16%), (vii) sweating (16%), and (viii) sore throat (3%). The incubation period observed for all cases ranged from 0.25 to 24 h, with a median of 2 h. The duration of illness ranged from 0.25 to 144 h, with a median estimate of 6 h.

A traceback was conducted, with the source of the outbreak determined to be a processing plant in Pennsylvania. The distributor in Pennsylvania packaged the refried beans specifically for the chain establishment where the outbreak occurred. The establishments where the outbreak occurred received 5 lb trays of pre-cooked, sealed, and frozen refried beans from the production/packaging facility. The refried beans would undergo cooking and a hot hold prior to consumption at the establishments where the outbreak occurred. It was determined that the refried beans were contaminated prior to preparation at the chain establishment.

Stool samples from suspect cases were cultured on MYP agar and *B. cereus*-like colonies were isolated from seven stool samples. Additionally, *B. cereus-*like colonies were isolated from nine food samples that were collected from five restaurants. In total, seven isolates from stool samples and 26 isolates from foods were confirmed to belong to the *B. cereus* group using standard microbiological methods. Isolates that were large Gram-positive rods, beta-hemolytic, and motile were presumptively identified as *B. cereus*-like. Additionally, spore staining was performed to test for the presence of parasporal crystals associated with *B. thuringiensis*, for which all isolates were negative. All 33 *B. cereus* group isolates underwent preliminary molecular characterization by Sanger sequencing of *rpoB*, which revealed two distinct allelic types belonging to phylogenetic groups III (*rpoB* allelic type AT 125) and IV (AT 92).

### WGS Confirms Presence of Multiple *B. cereus* Group Species Represented Among Outbreak Strains

*rpoB* allelic types (ATs) assigned *in silico* were identical to those obtained using Sanger sequencing for all 33 isolates (Table 3). *panC* group assignment confirmed the presence of *B. cereus s.l*. isolates from multiple phylogenetic groups (Table 3), with *panC* group III (*n* = 30) and *panC* group IV (*n* = 3) represented among the 33 isolates. *In silico* MLST further resolved the group IV isolates into two sequence types (STs): the two strains isolated from refried beans served at two different restaurants had identical STs, while the single human isolate belonging to group IV had a unique ST (Table 3). All 30 *panC* group III isolates belonged to ST 26, including the remaining six human clinical isolates (Table 3).

**Table 3:** List of outbreak isolates and corresponding metadata, single- and multi-locus sequence types, and species.

**Isolate name**	**Source (general)**	**Source (specific)**	**Collection date**	**Isolation date**	**Production date/batch^a^**	***panC* group^b^**	**MLST ST^c^**	***rpoB* AT^d^**	**Closest type strain (ANI)^e^**
FOOD_10_18_16_LFTOV_NA_R9-6400	Food	Leftovers	9-Oct-16	18-Oct-16	Unknown	III	26	125	*B. paranthracis* MN5 (97.5)
FOOD_10_18_16_LFTOV_NA_R9-6401	Food	Leftovers	9-Oct-16	18-Oct-16	Unknown	III	26	125	*B. paranthracis* MN5 (97.5)
FOOD_10_18_16_LFTOV_NA_R9-6402	Food	Leftovers	9-Oct-16	18-Oct-16	Unknown	III	26	125	*B. paranthracis* MN5 (97.5)
FOOD_10_19_16_RSNT1_1B_R9-6388	Food	Restaurant 1	6-Oct-16	19-Oct-16	1/B	III	26	125	*B. paranthracis* MN5 (97.5)
FOOD_10_19_16_RSNT1_1B_R9-6389	Food	Restaurant 1	6-Oct-16	19-Oct-16	1/B	III	26	125	*B. paranthracis* MN5 (97.5)
FOOD_10_19_16_RSNT1_1B_R9-6390	Food	Restaurant 1	6-Oct-16	19-Oct-16	1/B	III	26	125	*B. paranthracis* MN5 (97.5)
FOOD_10_19_16_RSNT1_1B_R9-6391	Food	Restaurant 1	6-Oct-16	19-Oct-16	1/B	III	26	125	*B. paranthracis* MN5 (97.5)
FOOD_10_19_16_RSNT1_2A_R9-6386	Food	Restaurant 1	6-Oct-16	19-Oct-16	2/A	III	26	125	*B. paranthracis* MN5 (97.5)
FOOD_10_19_16_RSNT1_2A_R9-6387	Food	Restaurant 1	6-Oct-16	19-Oct-16	2/A	III	26	125	*B. paranthracis* MN5 (97.5)
FOOD_10_19_16_RSNT1_2H_R9-6392	Food	Restaurant 1	6-Oct-16	19-Oct-16	2/H	III	26	125	*B. paranthracis* MN5 (97.5)
FOOD_10_19_16_RSNT1_2H_R9-6393	Food	Restaurant 1	6-Oct-16	19-Oct-16	2/H	III	26	125	*B. paranthracis* MN5 (97.5)
FOOD_10_19_16_RSNT1_2H_R9-6394	Food	Restaurant 1	6-Oct-16	19-Oct-16	2/H	III	26	125	*B. paranthracis* MN5 (97.5)
FOOD_10_19_16_RSNT1_2H_R9-6395	Food	Restaurant 1	6-Oct-16	19-Oct-16	2/H	III	26	125	*B. paranthracis* MN5 (97.5)
FOOD_10_19_16_RSNT1_2H_R9-6396	Food	Restaurant 1	6-Oct-16	19-Oct-16	2/H	III	26	125	*B. paranthracis* MN5 (97.5)
FOOD_10_19_16_RSNT2_2A_R9-6397	Food	Restaurant 2	6-Oct-16	19-Oct-16	2/A	III	26	125	*B. paranthracis* MN5 (97.5)
FOOD_10_19_16_RSNT2_2A_R9-6398	Food	Restaurant 2	6-Oct-16	19-Oct-16	2/A	III	26	125	*B. paranthracis* MN5 (97.5)
FOOD_10_19_16_RSNT2_2A_R9-6399	Food	Restaurant 2	6-Oct-16	19-Oct-16	2/A	III	26	125	*B. paranthracis* MN5 (97.6)
FOOD_10_19_16_RSNT3_1E_R9-6407	Food	Restaurant 3	6-Oct-16	19-Oct-16	1/E	III	26	125	*B. paranthracis* MN5 (97.5)
FOOD_10_19_16_RSNT3_2A_R9-6403	Food	Restaurant 3	6-Oct-16	19-Oct-16	2/A	III	26	125	*B. paranthracis* MN5 (97.5)
FOOD_10_19_16_RSNT3_2A_R9-6404	Food	Restaurant 3	6-Oct-16	19-Oct-16	2/A	III	26	125	*B. paranthracis* MN5 (97.5)
FOOD_10_19_16_RSNT3_2A_R9-6405	Food	Restaurant 3	6-Oct-16	19-Oct-16	2/A	III	26	125	*B. paranthracis* MN5 (97.5)
FOOD_10_19_16_RSNT4_2B_R9-6408	Food	Restaurant 4	6-Oct-16	19-Oct-16	2/B	III	26	125	*B. paranthracis* MN5 (97.5)
FOOD_10_19_16_RSNT4_2B_R9-6409	Food	Restaurant 4	6-Oct-16	19-Oct-16	2/B	III	26	125	*B. paranthracis* MN5 (97.5)
FOOD_10_19_16_RSNT5_1C_R9-6411	Food	Restaurant 5	6-Oct-16	19-Oct-16	1/C	III	26	125	*B. paranthracis* MN5 (97.5)
HUMN_10_18_16_FECAL_NA_R9-6384	Human	Feces	7-Oct-16	18-Oct-16	NA	III	26	125	*B. paranthracis* MN5 (97.6)
HUMN_10_18_16_FECAL_NA_R9-6385	Human	Feces	8-Oct-16	18-Oct-16	NA	III	26	125	*B. paranthracis* MN5 (97.5)
HUMN_10_18_16_FECAL_NA_R9-6412	Human	Feces	8-Oct-16	18-Oct-16	NA	III	26	125	*B. paranthracis* MN5 (97.5)
HUMN_10_19_16_FECAL_NA_R9-6381	Human	Feces	7-Oct-16	19-Oct-16	NA	III	26	125	*B. paranthracis* MN5 (97.5)
HUMN_10_19_16_FECAL_NA_R9-6382	Human	Feces	7-Oct-16	19-Oct-16	NA	III	26	125	*B. paranthracis* MN5 (97.5)
HUMN_10_19_16_FECAL_NA_R9-6383	Human	Feces	7-Oct-16	19-Oct-16	NA	III	26	125	*B. paranthracis* MN5 (97.5)
FOOD_10_19_16_RSNT3_1E_R9-6406	Food	Restaurant 3	6-Oct-16	19-Oct-16	1/E	IV	24	92	*B. cereus* ATCC 14579 (98.9)
FOOD_10_19_16_RSNT5_1C_R9-6410	Food	Restaurant 5	6-Oct-16	19-Oct-16	1/C	IV	24	92	*B. cereus* ATCC 14579 (98.9)
HUMN_10_26_16_FECAL_NA_R9-6413	Human	Feces	8-Oct-16	26-Oct-16	NA	IV	142	92	*B. cereus* ATCC 14579 (98.8)

a *Production date is designated by either 1 or 2; batch is one of A through H*.

b *panC group assigned in silico using BTyper 2.2.0*.

c *Multi-locus sequence typing (MLST) sequence type (ST) assigned in silico using BTyper 2.2.0*.

d *rpoB allelic type (AT) determined using Sanger sequencing and verified in silico using BTyper 2.2.0*.

e *ANI, average nucleotide identity calculated using FastANI*.

**Figure 1:**
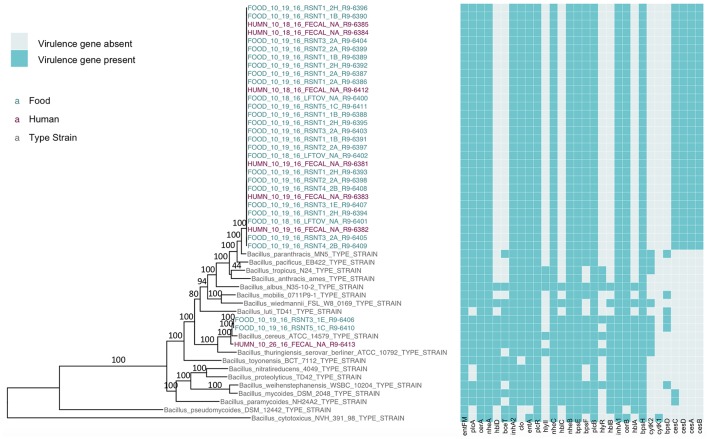
Maximum likelihood phylogeny of core SNPs identified in 33 isolates sequenced in conjunction with a *B. cereus* outbreak, as well as genomes of the 18 currently recognized *B. cereus* group species (shown in gray). Core SNPs were identified in all genomes using kSNP3. Heatmap corresponds to presence/absence of *B. cereus* group virulence genes detected in each sequence using BTyper. Tip labels in maroon and teal correspond to the seven human clinical isolates and 26 isolates from food sequenced in conjunction with this outbreak, respectively. Phylogeny is rooted at the midpoint, and branch labels correspond to bootstrap support percentages out of 500 replicates. Due to the short lengths and low bootstrap support (all values <10) of branches within the outbreak clade, bootstrap support percentages are not shown on branches within the outbreak clade.

The presence of isolates from multiple *B. cereus s.l*. phylogenetic groups, as suggested by the *rpoB, panC*, and MLST loci among isolates sequenced in conjunction with this outbreak, was confirmed using core SNPs detected in all outbreak isolates, as well as the genomes of 18 currently recognized *B. cereus* group species (Figure 1). The three isolates assigned to *panC* group IV using a 7-group scheme (Guinebretière et al., 2008) were most closely related to the *B. cereus s.s*. type strain (Figure 1). All three group IV *B. cereus* isolates possessed diarrheal toxin genes *hblABCD* and *cytK-2* at high identity and coverage (Figure 1), which code for enterotoxins hemolysin BL (Hbl) and cytotoxin K variant 2 (CytK-2), respectively. The 30 isolates assigned to *panC* group III, however, were most closely related to the type strain of *B. paranthracis* (Figure 1). Unlike the *B. paranthracis* type strain, all of the group III isolates investigated here were motile and possessed the *cesABCD* operon (Figure 1), which codes for emetic toxin-producing cereulide synthetase. In the case of isolate HUMN_10_18_16_FECAL_NA_R9-6384, *cesD* was split onto two contigs.

Based on average nucleotide identity (ANI) values, the three diarrheal group IV isolates were classified as *B. cereus s.s*. (ANI > 95; Table 3). The 30 emetic group III isolates from this outbreak, however, most closely resembled the type strain of *B. paranthracis* (ANI > 95; Table 3), indicating that the emetic group III and diarrheal group IV isolates from this outbreak are different *B. cereus* group species.

### Emetic and Diarrheal *B. cereus* Isolates Associated With the Foodborne Outbreak do Not Differ in Cytotoxicity

**Figure 2:**
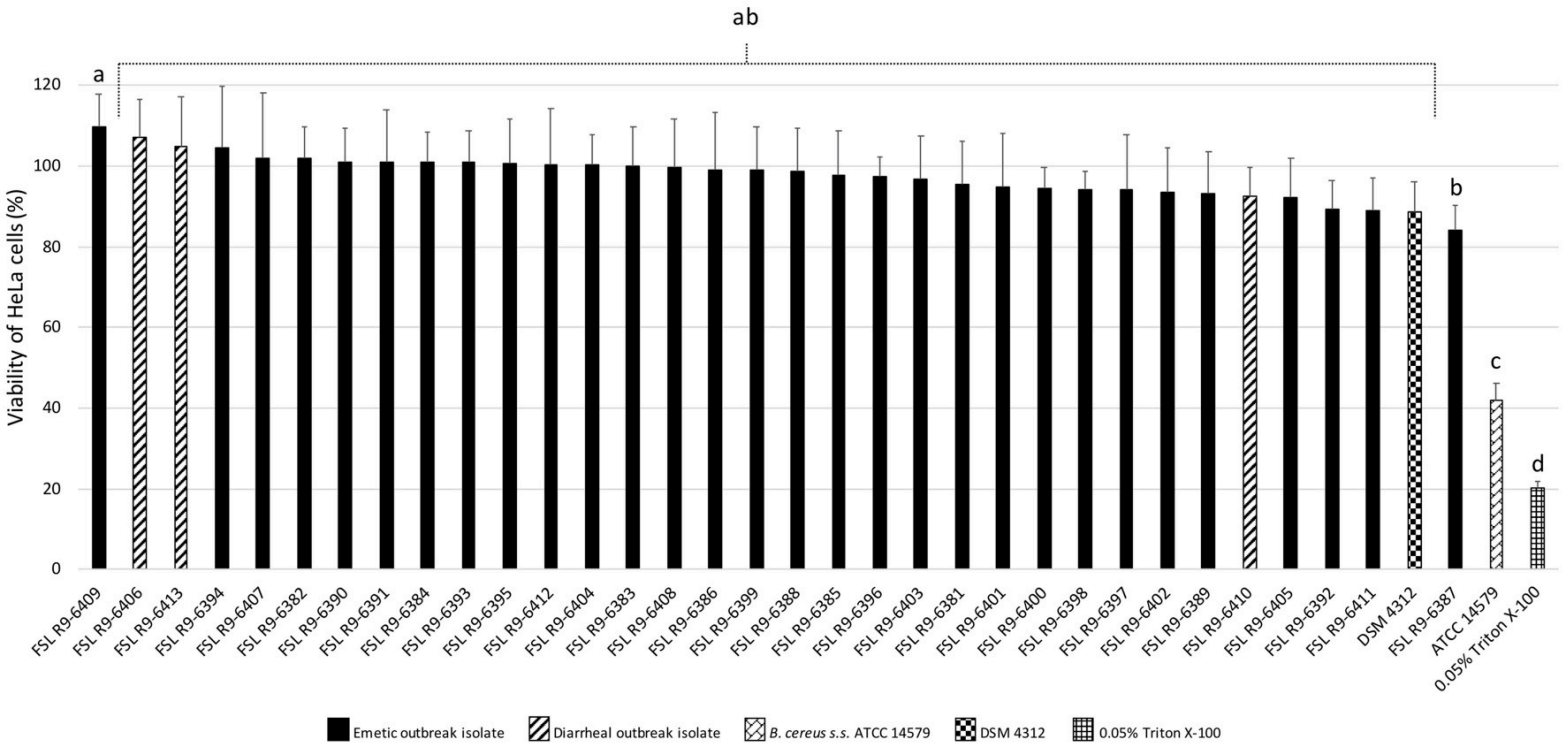
Percentage viability of HeLa cells when treated with supernatants of each isolate as determined by the WST-1 assay. Viability was calculated as ratio of corrected absorbance of solution when HeLa cells were treated with supernatants to the ratio of corrected absorbance of solution when HeLa cells were treated with BHI (i.e., negative control), converted to percentages. The columns represent the mean viabilities, while the error bars represent standard deviations for 12 technical replicates. Any two bars that do not share a common alphabetic character had significantly different percentage viability values (*P* < 0.05).

All three diarrheal strains isolated in conjunction with the outbreak (FSL R9-6406, FSL R9-6410, and FSL R9-6413) were found to produce Hbl, as well as non-hemolytic enterotoxin (Nhe). Characterization of six representatives of the emetic isolates tested (i.e., FSL R9-6381, FSL R9-6382, FSL R9-6384, FSL R9-6389, FSL R9-6395, and FSL R9-6399) revealed that they produced Nhe, but not Hbl. The supernatant of diarrheal *B. cereus s.s*. ATCC 14579 showed a stronger inhibitory effect on the viability of HeLa cells compared to supernatants of the 33 outbreak-associated isolates (Games-Howell *P* < 0.05; Figure 2). Furthermore, the viability of HeLa cells treated with 0.05% Triton X-100, the positive control, was significantly lower compared to viability of HeLa cells treated with bacterial supernatants (Games-Howell *P* < 0.05; Figure 2). Among all pairs of emetic isolates, only the viabilities of HeLa cells exposed to the supernatants of isolates FSL R9-6409 and FSL R9-6387 were found to differ (Games-Howell *P* < 0.05; Figure 2). The differences in HeLa cell viability after treatment with supernatants of these two emetic outbreak-associated strains are likely due to biological variability among replicates, as outbreak-associated emetic isolates were shown to be clonal (Figure 1). Taken together, the emetic group (represented by 30 emetic outbreak-associated isolates) had a mean cell viability of 97.5 ± 5.1%, while the diarrheal group (represented by three diarrheal outbreak-associated isolates) gave a mean cell viability of 101.4 ± 7.9%, as compared to the HeLa cells treated with BHI (i.e., negative control).

### Core SNPs Identified Among *B. cereus* Group Outbreak Isolates From Two Phylogenetic Groups Are Dependent on Variant Calling Pipeline and Reference Genome Selection

**Figure 3:**
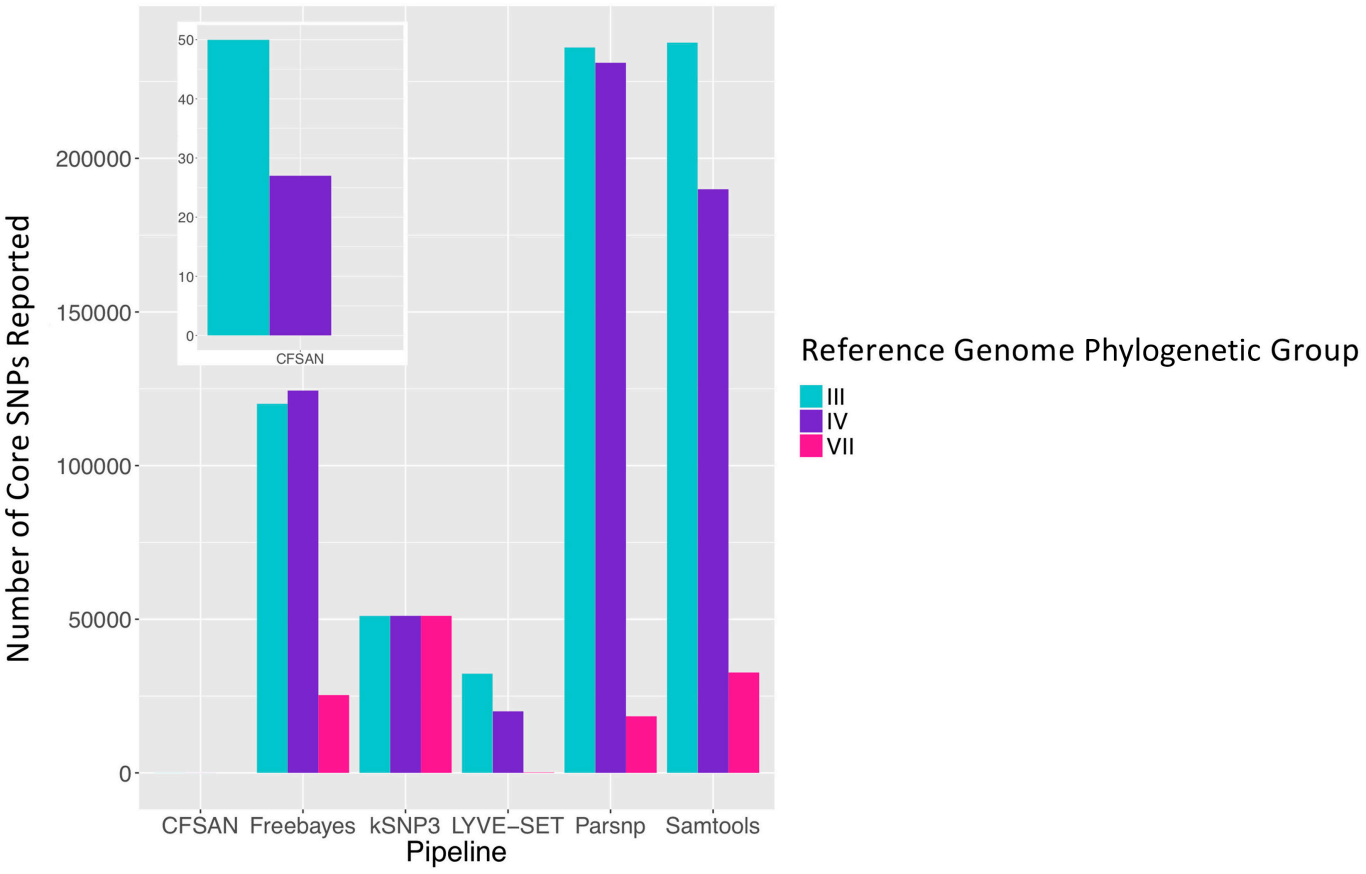
Number of core SNPs identified in 33 *B. cereus* group isolates from two phylogenetic groups (30 and 3 isolates from groups III and IV, respectively), sequenced in conjunction with a foodborne outbreak. Combinations of five reference-based variant calling pipelines and three reference genomes, as well as one reference-free SNP calling method (kSNP3), were tested.

**Figure 4:**
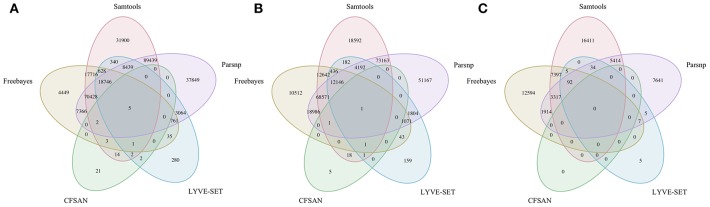
Comparison of core SNP positions reported by five reference-based variant-calling pipelines for 33 *B. cereus* group strains isolated in association with a foodborne outbreak, with the chromosomes of **(A)** *B. cereus* AH187 (group III), **(B)** *B. cereus s.s*. ATCC 14579 (group IV), and **(C)** *B. cytotoxicus* NVH 391-98 (group VII) used as reference genomes. Ellipses represent each pipeline.

To simulate a scenario in which genomes from a *B. cereus* outbreak spanning multiple phylogenetic groups were analyzed in aggregate, core SNPs were identified in all 33 outbreak isolates from groups III and IV (*n* = 30 and three isolates, respectively) using (i) combinations of five reference-based variant calling pipelines (Table 1) and three different reference genomes (Table 2) and (ii) a reference-free SNP calling method (Table 1). When genomes from all 33 isolates were analyzed together, the number of core SNPs identified by each pipeline and reference combination varied by up to several orders of magnitude (Figure 3), often with little agreement between pipelines in terms of the core SNPs they reported (Figure 4). Independent of reference genome, the CFSAN pipeline was the most conservative, consistently identifying the fewest number of core SNPs when all 33 isolates were queried in aggregate (50, 27, and 0 core SNPs using reference genomes from groups III, IV, and VII, respectively) (Figure 3). This can be contrasted with the Samtools, Freebayes, and Parsnp pipelines, which produced upwards of 100,000 core SNPs when the selected reference genome was a member of one of the groups being queried in the outbreak isolate set (group III and IV; Figure 3). In cases where a distant genome was used as the reference (group VII *B. cytotoxicus* type strain chromosome), all reference-based pipelines reported fewer core SNPs than kSNP3's reference-free *k-*mer based SNP calling approach (Figure 3).

### Choice of Variant Calling Pipeline Has Greater Influence on Core SNP Identification Than Choice of Closely Related Closed or Draft Reference Genome for Emetic Group III *B. cereus* Group Isolates

**Figure 5:**
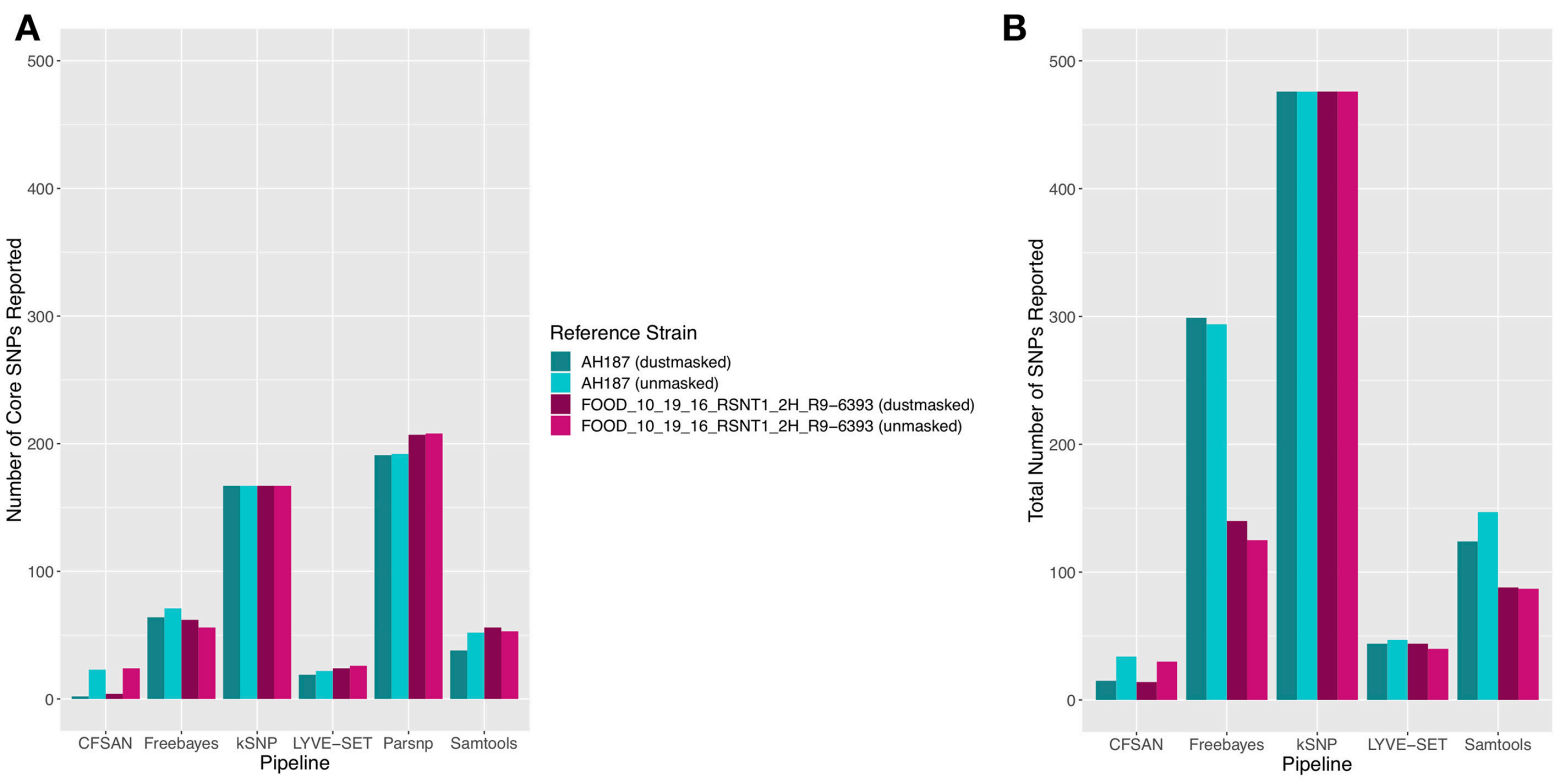
**(A)** Number of core SNPs and **(B)** total number of SNPs identified in 30 emetic *B. cereus* group III strains isolated in association with a foodborne outbreak. Combinations of **(A)** five and **(B)** four reference-based variant calling pipelines and two reference genomes (either dustmasked or unmasked) were tested, along with one reference-free SNP calling method (kSNP3). Because the Parsnp pipeline reports core SNPs by definition, it was excluded from Figure 5B (total SNPs). For quantification of the total number of SNPs (Figure 5B), all sites with more than one unique character were counted.

**Figure 6:**
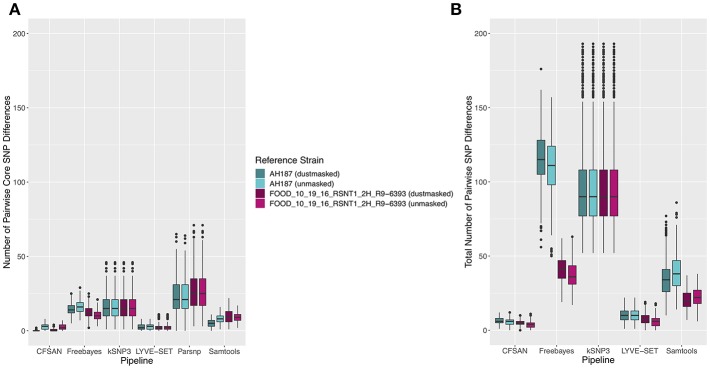
Ranges of pairwise **(A)** core SNP differences and **(B)** total SNP differences between 30 emetic group III *B. cereus* group strains isolated in conjunction with a foodborne outbreak. Combinations of **(A)** five and **(B)** four reference-based variant calling pipelines and two reference genomes (either dustmasked or unmasked), as well as one reference-free SNP calling method (kSNP3) were tested. Lower and upper box hinges correspond to the first and third quartiles, respectively. Lower and upper whiskers extend from the hinge to the smallest and largest values no more distant than 1.5 times the interquartile range from the hinge, respectively. Points represent pairwise distances that fall beyond the ends of the whiskers. Because the Parsnp pipeline reports core SNPs by definition, it was excluded from Figure 6B (pairwise differences in total SNPs). For quantification of pairwise differences in the total number of SNPs (Figure 6B), all sites with more than one unique character were included.

**Figure 7:**
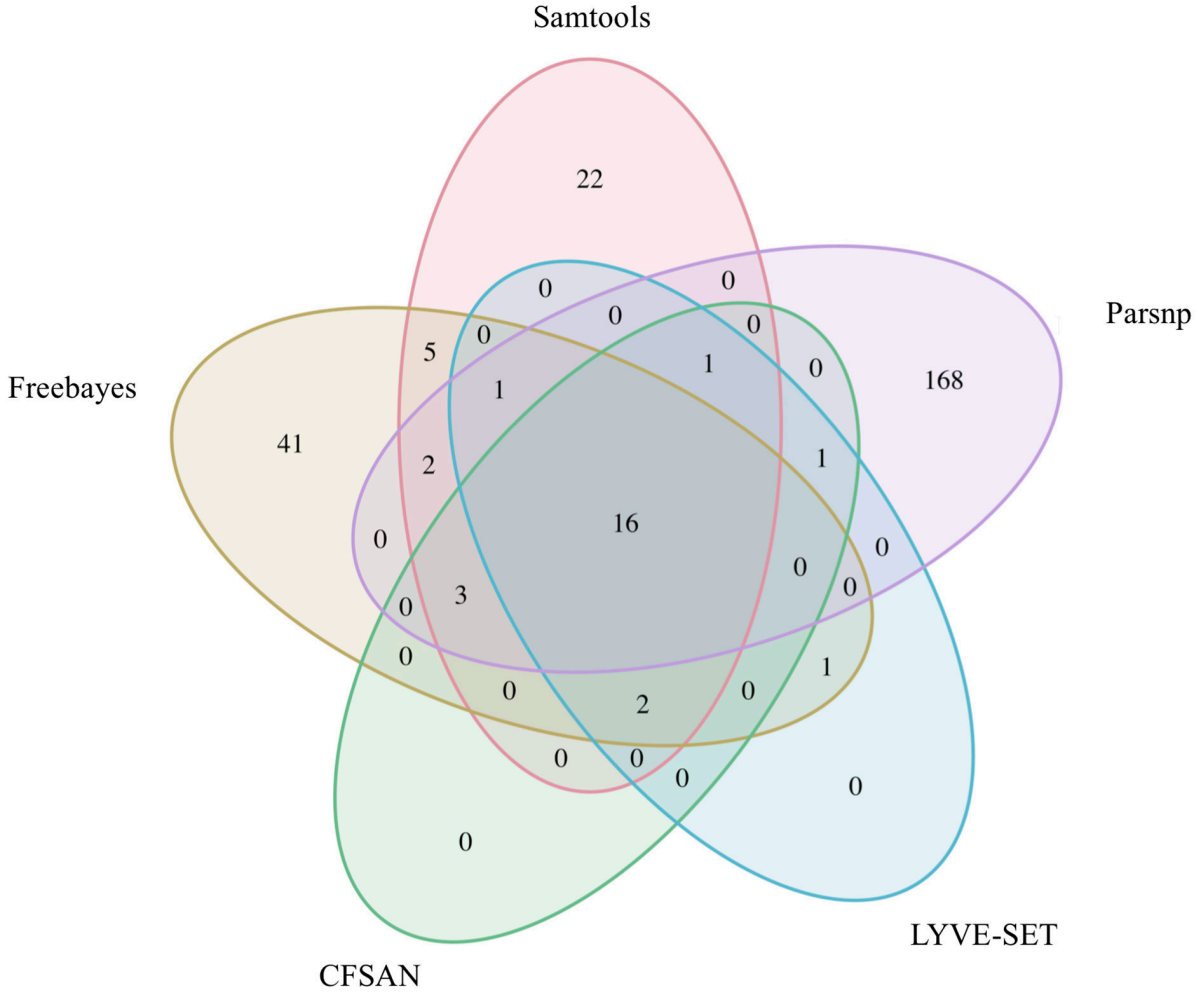
Comparison of core SNP positions reported by five variant-calling pipelines for 30 emetic group III *B. cereus* group outbreak isolates. Ellipses represent each pipeline, all of which used the chromosome of emetic group III *B. cereus* AH187 as a reference for variant calling.

The 30 emetic group III isolates were queried in the absence of their group IV counterparts using combinations of five reference-based variant calling pipelines (Table 1) and two reference genomes (the closed chromosome of *B. cereus* AH187, with and without dustmasking, and contigs of one of the isolates identified in this outbreak, with and without dustmasking; Table 2) and one reference-free SNP calling method (Table 1). In this scenario, the choice of variant calling pipeline had a greater effect on the number of core SNPs obtained than the choice of reference genome, as both reference genomes possessed the same virulence gene profile (virulotype), *rpoB* AT, *panC* group, MLST sequence type, and were of the same species (*B. paranthracis*; ANI > 95%) as the 30 emetic isolates (Figure 5A). Congruent with this, the number of pairwise core SNP differences between emetic isolates sequenced in this outbreak varied more with the selection of variant calling pipeline than with reference genome (Figure 6A). When the unmasked closed chromosome of *B. cereus* AH187 was used as a reference, pairwise core SNP differences among emetic isolates from this outbreak ranged from 0 to 8 (mean of 2.9; CFSAN), 7 to 29 (mean of 16.1; Freebayes), 0 to 8 (mean of 2.8; LYVE-SET), 0 to 64 (mean of 23.6; Parsnp), and 1 to 16 SNPs (mean of 8.2; Samtools) (Figure 5A). Using the reference-free kSNP3 pipeline, this range was 1–46 SNPs (mean of 16.7; Figure 5A). The CFSAN and LYVE-SET pipelines produced nearly identical results in terms of the number and identity of the core SNPs called (23 and 22 SNPs, respectively, 20 of which were detected by both pipelines; Figure 7), as well as the topologies of the phylogenies those SNPs produced: all CFSAN and LYVE-SET phylogenies were more similar to each other than what would be expected by chance (Table 4 and Supplementary Table S4). Additionally, the two methods that relied on assembled genomes rather than short reads for SNP calling (kSNP3 and Parsnp) produced the greatest numbers of core SNPs (Figure 5A).

**Table 4:** Maximum likelihood phylogenies of 30 emetic group III outbreak isolates considered to be more topologically similar than would be expected by chance (*P* < 0.05)^a^.

**Reference phylogeny^b^**	**Query phylogeny^b^**	**Corrected *P-*value^c^**
AH187_CFSAN_NOdust_all	AH187_CFSAN_NOdust_core	0
AH187_CFSAN_NOdust_all	AH187_LYVE-SET_NOdust_all	0
AH187_CFSAN_NOdust_all	AH187_LYVE-SET_NOdust_core	0.0171
AH187_CFSAN_NOdust_all	AH187_LYVE-SET_YESdust_all	0
AH187_CFSAN_NOdust_all	AH187_LYVE-SET_YESdust_core	0.0171
AH187_CFSAN_NOdust_core	AH187_LYVE-SET_NOdust_all	0
AH187_CFSAN_NOdust_core	AH187_LYVE-SET_NOdust_core	0.0171
AH187_CFSAN_NOdust_core	AH187_LYVE-SET_YESdust_all	0
AH187_CFSAN_NOdust_core	AH187_LYVE-SET_YESdust_core	0.0171
AH187_Freebayes_NOdust_core	AH187_Freebayes_YESdust_core	0.0342
AH187_LYVE-SET_NOdust_all	AH187_LYVE-SET_NOdust_core	0.0171
AH187_LYVE-SET_NOdust_all	AH187_LYVE-SET_YESdust_all	0
AH187_LYVE-SET_NOdust_all	AH187_LYVE-SET_YESdust_core	0.0171
AH187_LYVE-SET_NOdust_core	AH187_LYVE-SET_YESdust_core	0
AH187_LYVE-SET_YESdust_all	AH187_LYVE-SET_YESdust_core	0.0171
AH187_Parsnp_NOdust_core	AH187_Parsnp_YESdust_core	0.0171

a *Obtained from pairwise tests of tree topologies using a Z test based on the Kendall-Colijn metric (Kendall and Colijn, 2015; Katz et al., 2017); see Supplementary Table S4 for full table of comparisons*.

b *Names of reference and query phylogenies denote reference genome (“AH187” for reference-based pipelines, “NOREF” for reference-free kSNP pipeline), pipeline (“CFSAN,” “Freebayes,” “kSNP,” “LYVE-SET,” “Parsnp,” or “Samtools”), reference genome masking (“NOdust” for an unmasked reference genome, “YESdust” for a dustmasked reference genome, or “NAdust” for reference-free kSNP pipeline, for which dustmasking is not applicable), and SNPs used to construct the phylogeny (“core” for core SNPs, or “all” for core and accessory SNPs), separated by an underscore (“_”)*.

c *Bonferroni-corrected P-values for all tests that were significant at the α = 0.05 level*.

Within the emetic group III isolates associated with this outbreak, a total of 32 core SNPs were identified by two or more of the reference-based variant calling pipelines when the unmasked *B. cereus* AH187 genome was used as a reference, half of which were identified by all five pipelines (Figure 7). Out of these 32 SNPs, 23 were identified in protein coding genes, 14 of which produced non-synonymous amino acid changes (Supplementary Table S5). Genes with non-synonymous changes were involved in molybdopterin biosynthesis (WP_000544623.1), proteolysis (WP_000215096.1 and WP_000857793.1), chitin binding (WP_000795732.1), iron-hydroxamate transport (WP_000728195.1), DNA repair (WP_000947749.1 and WP_000867556.1), DNA replication (WP_000867556.1 and WP_000435993.1), protein transport and insertion into the membrane (WP_000727745.1), and glyoxylase/bleomycin resistance (WP_000800664.1).

In addition to detecting core SNPs in the genomes of the 30 emetic group III isolates, total (core and accessory) SNPs were detected in the 30 emetic group III genomes using combinations of four reference-based variant calling pipelines (Parsnp, which only reports core SNPs, was excluded; Table 1) and two reference genomes (the closed chromosome of *B. cereus* AH187 and contigs of one of the isolates identified in this outbreak, with and without dustmasking; Table 2) and one reference-free SNP calling method (Table 1). When total SNPs were accounted for, rather than solely core SNPs, all pipeline/reference genome combinations showed increases in the number of SNPs detected and the range of pairwise SNP differences between genomes (Figures 5B, 6B). Whether the addition of accessory SNPs translated into a significant difference in phylogenetic topology, however, depended on the variant calling pipeline used. When the *B. cereus* AH187 closed chromosome was used as a reference, SNPs detected using the LYVE-SET pipeline produced phylogenies considered to be more topologically similar than would be expected by chance (Kendall-Colijn test *P* < 0.05), regardless of whether core SNPs or total SNPs were used to construct the phylogeny, and regardless of whether the *B. cereus* AH187 reference genome was dustmasked or not (Table 4 and Supplementary Table S4). Additionally, all phylogenies produced using the LYVE-SET pipeline and the *B. cereus* AH187 reference genome (i.e., each combination of core SNPs, total SNPs, dustmasked reference, and unmasked reference) were topologically similar to those produced using the CFSAN pipeline and the unmasked *B. cereus* AH187 reference genome, regardless of whether all SNPs were included or solely core SNPs (Table 4 and Supplementary Table S4). Other topologically similar phylogeny pairs included phylogenies constructed using (i) core SNPs identified with Freebayes, regardless of whether a dustmasked reference genome was used or not, and (ii) core SNPs identified with Parsnp, regardless of whether a dustmasked reference was used or not (Kendall-Colijn test *P* < 0.05; Table 4 and Supplementary Table S4).

### Phylogenies Constructed Using Core SNPs Identified in 55 Emetic ST 26 *B. cereus* Genomes by kSNP3 and Parsnp Yield Similar Topologies

**Figure 8:**
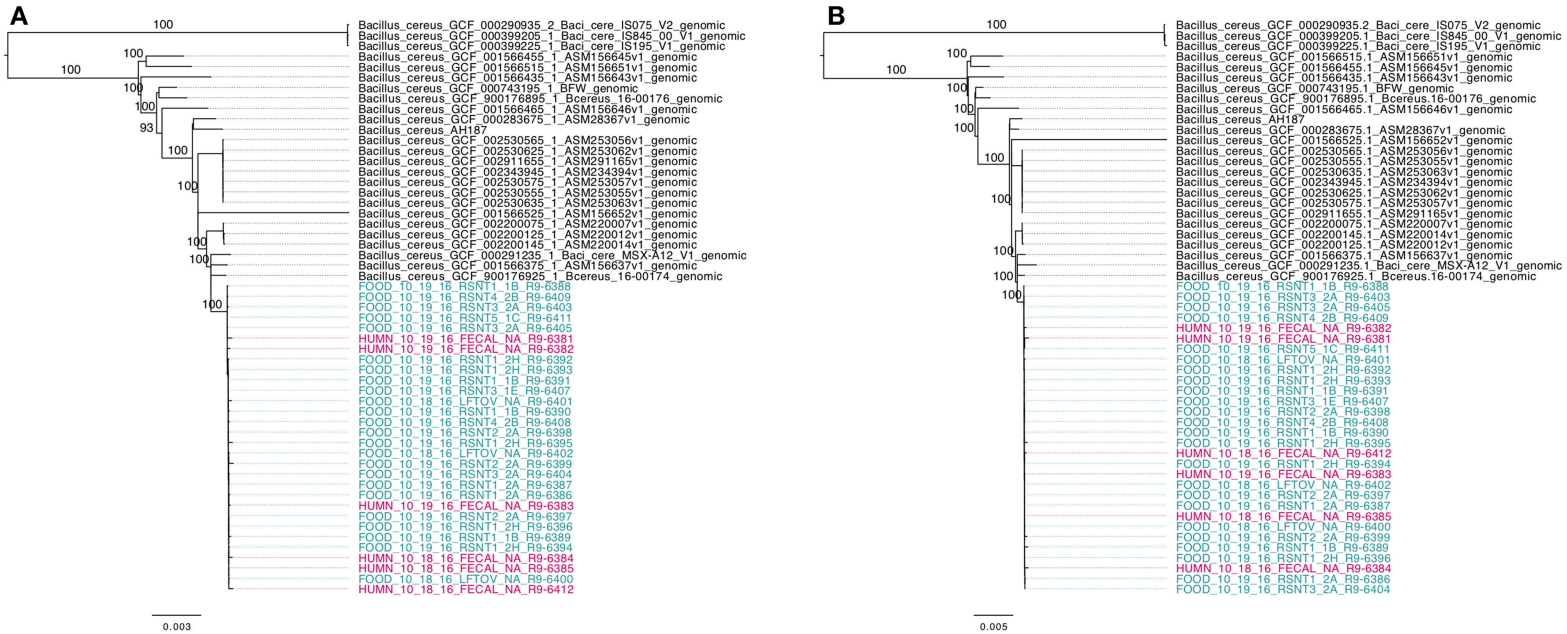
Maximum likelihood phylogenies of 30 emetic group III isolates (ST 26) sequenced in conjunction with a *B. cereus* outbreak, as well as all other emetic group III ST 26 genomes available in NCBI (*n* = 25; shown in black). Trees were constructed using core SNPs identified using **(A)** kSNP3 or **(B)** Parsnp. Tip labels in maroon and teal correspond to the six human clinical isolates and 24 isolates from food sequenced in conjunction with this outbreak, respectively. Branch labels correspond to bootstrap support percentages out of 1,000 replicates. Due to the short lengths and low bootstrap support of branches within the outbreak clade, bootstrap support percentages are not shown on branches within the outbreak clade.

To compare the 30 emetic strains from this outbreak to other emetic group III isolates, all emetic group III assembled genomes with ST 26 were downloaded from NCBI. This produced a total of 55 emetic group III isolates with ST 26 (30 isolates from this outbreak and 25 from NCBI RefSeq). Among the 55 emetic ST 26 genomes, Parsnp identified almost twice as many core SNPs as kSNP3 (4,597 and 2,593 core SNPs, respectively). However, the topologies of phylogenies produced using the core SNPs identified by each pipeline were found to be more similar than would be expected by chance (Kendall-Colijn test *P* < 0.05; Figure 8).

Based on pairwise core SNP differences, the publicly available genomes showed greater variability than the outbreak isolates described here, regardless of whether kSNP3 or Parsnp was used for variant calling (ANOVA-like permutation test *P* < 0.05; Supplementary Figure S1). Pairwise core SNP differences of the 30 emetic group III isolates from this outbreak ranged from 0 to 25 SNPs (mean of 8.3) and 0 to 44 SNPs (mean of 11.9) when the kSNP3 and Parsnp pipelines were used, respectively (Supplementary Figure S1). For external ST 26 isolates not associated with this outbreak, pairwise core SNP differences ranged from 0 to 1,474 SNPs (mean of 425.7) and 0 to 3,111 SNPs (mean of 828.3) when kSNP3 and Parsnp were used, respectively (Supplementary Figure S1). Between these two groups (the 30 emetic isolates from this outbreak and the 25 external emetic ST 26 isolates), pairwise core SNP differences ranged from 73 to 1,258 SNPs (mean of 301.7; kSNP3) and 74 to 2,709 SNPs (mean of 528.0; Parsnp) (Supplementary Figure S1). Reflecting this, the average of the ranks of pairwise SNP distances within emetic isolates from this outbreak was less than the average of the ranks of pairwise SNP distances between the emetic isolates from this outbreak and the external ST 26 isolates (ANOSIM *P* < 0.05). This is likely a result of the differences in variance between the outbreak and external ST 26 isolates, as supported by the results of the ANOVA-like permutation test (Anderson and Walsh, 2013).

## Discussion

While *B. cereus* causes a considerable number of foodborne illness cases annually, outbreaks are rarely investigated with the methodological rigor (e.g., use of WGS) that is increasingly used for surveillance and outbreak investigations targeting other foodborne pathogens. A specific challenge in the U.S. is that, unlike for some other diseases, disease cases caused by *B. cereus* are typically not reportable, even though foodborne illnesses, regardless of etiology, are reportable in some states, including NY. This, combined with the typically mild course of *B. cereus* infection, means that human *B. cereus* isolates are rarely available for WGS. Furthermore, even if clinical *B. cereus* group isolates are available, WGS may not be used for isolate characterization in cases where infections are mild. Due to the availability of *B. cereus* isolates for seven human cases, the outbreak reported here presented a unique opportunity to pilot the use of WGS for investigation of *B. cereus* outbreaks. The data and approaches presented here will not only facilitate future investigation of other *B. cereus* outbreaks but will also help with application of WGS for investigation of other foodborne disease outbreaks where limited reference WGS data and information on genomic diversity are available.

### Addressing the Microbiological and Epidemiological Challenges Associated With Determining the Causative Agent of an Emetic Foodborne Outbreak

The agar MYP used for isolation of strains from food and human clinical samples in the outbreak reported here is one of the two selective differential agars recommended in the FDA BAM protocol for the isolation of *B. cereus* group strains (Tallent et al., 2012b). The second recommended agar, Bacara, has been shown to be more selective and more effective in suppressing the growth of other Gram-positive microorganisms that may be present in tested samples (e.g., other *Bacillus* species, *Listeria, Staphylococcus*) (Tallent et al., 2012a; Kabir et al., 2017). Since Bacara medium has a proprietary formula and cannot be purchased in a dehydrated powder form (Tallent et al., 2012b), it is less likely to be readily available for use in labs that do not routinely test for *B. cereus* group species. Use of both types of media may increase the success of *B. cereus* group isolation from food and clinical samples, especially isolation of emetic strains (Ehling-Schulz et al., 2005; Ceuppens et al., 2013). Furthermore, the isolation of *B. cereus* group strains associated with this outbreak was carried out at 37°C, which is higher than the temperature of 30°C that is recommended by the FDA BAM (Tallent et al., 2012b). Nevertheless, while incubation at this temperature may inhibit the growth of psychrotolerant species of the *B. cereus* group (e.g., *B. weihenstephanensis*), it is not expected to interfere with the isolation of *B. cereus* group strains that are able to grow at human body temperature and cause toxicoinfection. It is also not expected to compromise isolation of emetic isolates with the capacity to cause intoxication, as emetic strains have been previously found primarily in phylogenetic group III, which does not contain psychrotolerant strains (Carroll et al., 2017). Overall, use of both types of isolation media and a moderate incubation temperature of 30°C may minimize the isolation bias.

While the isolation of *B. cereus* group strains from food and clinical samples is essential for linking them to a potential foodborne outbreak, further information is needed to definitively prove that an outbreak was caused by *B. cereus*. Emetic disease caused by members of the *B. cereus* group can be attributed to the production of the highly heat- and pH-resistant toxin cereulide in food prior to ingestion (Ehling-Schulz et al., 2004, 2015; Stenfors Arnesen et al., 2008). Because cereulide is produced within the food matrix itself, prior to consumption, the mere presence of emetic *B. cereus* group strains in food or human clinical samples cannot definitively prove that an outbreak was caused by a member of the *B. cereus* group; rather, the presence of cereulide itself is essential for linking food and clinical samples to an outbreak with high confidence (Andersson et al., 2004; Stenfors Arnesen et al., 2008). For this outbreak, the presence of cereulide in food and human clinical samples linked to the outbreak was not assessed, as testing for cereulide is not currently included in the BAM protocol as a routine method for the detection and enumeration of *B. cereus* in food. Ergo, there is no definitive proof that the outbreak was caused by cereulide-producing emetic group III *B. cereus* and not a similar foodborne pathogen (e.g., enterotoxins produced by *Staphylococcus aureus*, which manifest in similar symptoms to those associated with cereulide) (Messelhäusser et al., 2014). However, due to the presence of highly clonal, *ces-*positive group III ST 26 *B. cereus* group isolates among food and clinical samples linked to the outbreak, as well as epidemiological data that support this, the emetic strain is the most probable causative agent. While it is not currently included in the BAM protocol for *B. cereus* isolation (Tallent et al., 2012b), testing for the presence of cereulide in food and clinical samples linked to potential outbreaks caused by emetic *B. cereus* can aid in providing a definitive link between illness and causative agent.

### Considerations for Addressing the Unique Challenges Associated With Characterization of Foodborne Outbreaks Linked to the *B. cereus* Group Using WGS

In *B. cereus* outbreaks, interpretation of WGS data can be challenging, especially in cases where strains of multiple closely related species or subtypes appear to be associated with an outbreak. *B. cereus* outbreaks—particularly emetic outbreaks caused by cereulide-producing *B. cereus* group isolates—are often associated with improper handling of food (e.g., temperature abuse) (Ehling-Schulz et al., 2004; Stenfors Arnesen et al., 2008). This, and their ubiquitous presence in the environment, make it important to consider the possibility of a multi-strain or multi-species outbreak in addition to a single-source outbreak caused by a single strain. In the outbreak characterized here, *B. cereus* group strains from two phylogenetic groups, III and IV, were isolated from both human clinical stool samples, as well as refried beans linked to the outbreak. The separation of outbreak-related isolates into three diarrheal group IV isolates (representing two distinct STs) and 30 emetic isolates may be explained by one of the following scenarios: (i) the outbreak was caused by refried beans contaminated with multiple *B. cereus* group species (isolates from groups III and IV), both of which caused illness in humans, (ii) in addition to housing emetic outbreak strains that belonged to group III, samples of refried beans and patient stool samples harbored group IV *B. cereus s.l*. isolates that were not part of the outbreak but were incidentally isolated from stool and food samples, or (iii) a subset of patient stool samples and food samples did not harbor *B. cereus s.l*. group III isolates belonging to the outbreak, but did harbor group IV strains that were isolated and sequenced. In order to determine which of these scenarios explains the presence of multiple *B. cereus* group species among isolates sequenced in conjunction with a foodborne outbreak, additional epidemiological and microbiological data are needed.

Valuable metrics for inclusion/exclusion of *B. cereus* group cases in a foodborne outbreak include patient exposure, patient symptoms (e.g., vomiting, diarrhea, onset and duration of illness), levels of *B. cereus* present in implicated food and patient samples (CFU/g or CFU/ml), cytotoxicity of isolates, and the approach used to select bacterial colonies to undergo WGS (Glasset et al., 2016). However, some of these data may be more valuable than others. In their characterization of 564 *B. cereus* group strains associated with 140 strong-evidence foodborne outbreaks in France between 2007 and 2014, Glasset et al. (2016) found that patient symptoms could not be associated with the presence of emetic and diarrheal strains. More than half (57%) of the *B. cereus* outbreaks queried in their study included patients exhibiting both emetic and diarrheal symptoms. Similar results were observed here, as emetic and diarrheal symptoms were reported in 88 and 38% of cases, respectively, with both vomiting and diarrhea reported by multiple patients. All emetic isolates associated with this outbreak carried *nhe* genes and also produced Nhe enterotoxin, as determined using the immunoassay. While it has been proposed that a combination of emetic and diarrheal symptoms may be due to the fact that emetic group III isolates have been shown to produce diarrheal enterotoxin Nhe at high levels (Glasset et al., 2016), incongruences between isolate virulotype and patient symptoms may still exist. Importantly, this indicates the need for further investigation of factors affecting the expression of *B. cereus* group virulence genes, as well as their potential synergistic activities (Doll et al., 2013).

Another metric that can be used for determining whether *B. cereus* group isolates are part of an outbreak or not is the level of *B. cereus* present in the implicated food. Like patient symptoms, *B. cereus* counts from implicated foods may aid in an outbreak investigation, but likely cannot definitively prove whether an isolate is part of an outbreak or not. For example, outbreaks caused by implicated foods with *B. cereus* counts of <10^3^ CFU/g and as low as 400 CFU/g for diarrheal and emetic diseases, respectively, have been described (Glasset et al., 2016), despite levels of at least 10^5^ CFU/g often being detected in implicated foods (Stenfors Arnesen et al., 2008). The levels of *B. cereus* present in refried beans in the outbreak described here were not determined. However, like patient symptoms, *B. cereus* count data may be a useful supplemental metric for investigating *B. cereus* group outbreaks in the future.

In addition to patient symptoms and pathogen load in the food, incubation period can be used to determine whether an isolate is part of an outbreak or not, as it is significantly shorter for emetic strains than diarrheal strains (Ehling-Schulz et al., 2004; Stenfors Arnesen et al., 2008; Glasset et al., 2016). In the outbreak described here, the patient from which a non-emetic group IV *B. cereus* group strain was isolated reported an incubation time of 1 h, the lowest incubation time of all seven confirmed human clinical cases. However, this is still within the observed range of incubation times for emetic *B. cereus* disease (0.5–6 h) (Stenfors Arnesen et al., 2008). Although no emetic group III *B. cereus s.l*. strain was isolated from the clinical sample, it is possible that the patient could have been intoxicated with cereulide produced in the food by the emetic *B. cereus* strain that caused the outbreak. However, it is also possible that a pathogen which causes similar symptoms to foodborne illness caused by emetic *B. cereus* was responsible for the patient's illness (e.g., *Staphylococcus aureus*).

Lastly, cytotoxicity data may also be leveraged to include/exclude outbreak-associated *B. cereus* group isolates. In the outbreak described here, the patient from which a non-emetic group IV *B. cereus* group strain was isolated reported vomiting and nausea and no diarrheal symptoms, despite the clinical isolate's possession of multiple diarrheal toxin genes and no emetic toxin genes. This could suggest that the patient was intoxicated with the cereulide, but the isolate itself did not survive the passage through the patient's gastrointestinal tract, or that it survived in a low concentration that resulted in failure of isolation on MYP. It is also possible that our understanding of the specific virulence genes responsible for different *B. cereus-*associated disease symptoms is still incomplete and that the diarrheal isolate obtained from the clinical sample was in fact responsible for symptoms of vomiting and nausea. To further investigate this, we carried out immunoassay-based detection of Hbl and Nhe enterotoxins, as well as a WST-1 proliferation assay with HeLa cells exposed to bacterial supernatants presumably containing toxins. The results of Hbl and Nhe immunodetection and cytotoxicity revealed that diarrheal isolates only had mild detrimental effects on HeLa cell viability, despite the fact that they produced both hemolysin BL and non-hemolytic enterotoxins. This can be contrasted with the *B. cereus s.s*. type strain, which substantially reduced the viability of the HeLa cells.

For the outbreak described here, results obtained using a combination of microbiological, epidemiological, and bioinformatic methods indicate that hypothesis (i), in which the diarrheal strains were part of a multi-species outbreak, can likely be excluded. Evidence supporting the conclusion that the human clinical diarrheal isolate was not part of the outbreak described here include: (i) the emetic symptoms reported by the patient were incongruent with the virulotype of the isolate, (ii) the incubation time was typical for intoxication, (iii) the human clinical diarrheal isolate had a different MLST ST compared to all other isolates sequenced in this outbreak, and (iv) the human diarrheal isolate did not exhibit substantial cytotoxicity against HeLa cells (Figure 2). This may be due to the fact that this case was not part of the outbreak and was due to an infection or intoxication caused by another pathogen that leads to disease symptoms similar to *B. cereus* (e.g., *Staphylococcus aureus*), or that a group IV *B. cereus* strain was isolated and sequenced in lieu of the group III emetic outbreak isolate. There is limited evidence as to whether humans can be asymptomatic carriers of group IV *B. cereus* (Ghosh, 1978; Turnbull and Kramer, 1985), making it likely that isolation and sequencing of a group IV *B. cereus* strain could be due to the use of MYP agar as the sole selective agar, which has been shown to hinder detection of emetic *B. cereus* group isolates (Ehling-Schulz et al., 2005; Ceuppens et al., 2013). In future outbreaks, the use of additional selective media (e.g., Bacara agar), enrichment media, and isolation temperatures may aid in isolation of the causative *B. cereus* group strain.

While we have shown here that WGS data can be a valuable tool for characterizing *B. cereus* group isolates from a foodborne outbreak, our results also showcase the importance of supplementing WGS data with epidemiological and microbiological metadata to draw meaningful conclusions from *B. cereus* group genomic data. Furthermore, the availability of WGS and cytotoxicity data from a larger set of *B. cereus* isolates from symptomatic patients may also provide an opportunity to use comparative genomics approaches to further explore virulence genes that are linked to different disease outcomes in the future.

### Recommendations for Analyzing Illumina WGS Data From *B. cereus* Group Isolates Potentially Linked to a Foodborne Outbreak

WGS is being used increasingly to characterize isolates associated with foodborne disease cases and outbreaks, and rightfully so, as it offers the ability to characterize foodborne pathogens at unprecedented resolution, and it has been able to improve outbreak and cluster detection for numerous foodborne pathogens (Allard et al., 2017; Kovac et al., 2017; Moran-Gilad, 2017; Taboada et al., 2017), including *Salmonella enterica* (Taylor et al., 2015; Hoffmann et al., 2016; Gymoese et al., 2017), *Escherichia coli* (Grad et al., 2012; Holmes et al., 2015; Rusconi et al., 2016), and *Listeria monocytogenes* (Jackson et al., 2016; Kwong et al., 2016; Chen et al., 2017a,b; Moura et al., 2017). However, as demonstrated here and elsewhere, variant calling pipelines and the various mapping/alignment, SNP calling, and SNP filtering practices that they employ (e.g., removal of recombination and clustered SNPs) can influence the identification of SNPs in WGS data and, thus, the topology of a resulting phylogeny (Pightling et al., 2014, 2015; Croucher et al., 2015; Hwang et al., 2015; Katz et al., 2017; Sandmann et al., 2017). This can be particularly problematic for outbreak and cluster detection in bacterial pathogen surveillance: pairwise SNP thresholds are currently widely used to make initial decisions regarding the inclusion or exclusion of isolates in a given outbreak (Taylor et al., 2015; Gymoese et al., 2017; Mair-Jenkins et al., 2017; McCloskey and Poon, 2017; Walker et al., 2018). In such scenarios, just a few SNPs can be the deciding factor in whether a bacterial pathogen is included or excluded as part of an outbreak or cluster (Katz et al., 2017), rendering the choice of variant calling method as non-trivial. Furthermore, choosing an appropriate variant calling pipeline can be particularly challenging for pathogens where there are limited data and expertise with WGS, as is currently the case with *B. cereus*.

As demonstrated here, the choice of variant calling pipeline can greatly influence the number of core SNPs identified in *B. cereus* group isolates associated with a foodborne outbreak. In the case of a multi-group outbreak, this effect can be magnified. Naively calling variants in isolates that span multiple *B. cereus s.l*. phylogenetic groups in aggregate can lead to orders of magnitudes of difference in the number of core SNPs identified by different variant calling pipelines/reference genome combinations. In a multi-group outbreak scenario, it is essential to note that one is effectively dealing with genomic data from *multiple species* (i.e., ANI < 95), making it impossible to find a reference genome that is closely related to all isolates in a putative outbreak. In the case of some reference-based pipelines that are specifically tailored to identify variants in bacterial isolates from outbreaks (e.g., CFSAN, which is not suited for bacteria differing by more than a few hundred SNPs), calling variants in multiple groups or within a distant reference genome is inappropriate (Davis et al., 2015). Thus, querying outbreak isolates from multiple groups in aggregate using reference-based variant calling methods should be avoided. Furthermore, the results presented here showcase the value of employing single- and/or multi-locus typing approaches prior to variant calling, either via Sanger sequencing or *in silico* using tools, such as BTyper, as they can aid the design of downstream bioinformatics analyses, including reference genome selection and data partitioning by phylogenetic group.

When the three phylogenetic group IV isolates were excluded from analyses, leaving only the emetic group III isolates, the selection of reference genome caused fewer core SNP discrepancies than choice of variant calling pipeline, provided the reference genome was “similar” to the genomes analyzed. While the selection of a reference genome for reference-based variant calling is not trivial (Pightling et al., 2014; Olson et al., 2015), reference-based variant calling using a closed chromosome (*B. cereus* AH187) and a draft genome (FOOD_10_19_16_RSNT1_2H_R9-6393) from two isolates that were closely related to, or among the emetic group III isolates sequenced in this outbreak produced nearly identical results in terms of the number and identity of core SNPs detected. Both reference genomes were identical to the emetic group III outbreak isolates sequenced here in terms of *panC* group, *rpoB* AT, MLST ST, and virulotype. Additionally, the closed chromosome and draft genome had ANI values of >99.8 and 99.9, respectively, relative to all emetic group III outbreak isolates in this study, which can be considered highly similar. Comparable findings have been observed in analyses of *Salmonella enterica* serovar Heidelberg WGS data (Usongo et al., 2018), suggesting that either closed genomes or high-quality draft genomes are adequate for reference-based SNP calling, provided both are similar enough to the outbreak strains being queried. While the thresholds at which reference genomes become “similar enough” and of sufficient quality for reference-based SNP calling for outbreak detection warrant further investigation, we have demonstrated here that, for emetic group III ST 26 *B. cereus* group genomes, the publicly available closed chromosome of *B. cereus* AH187 can serve as an adequate standard.

With regard to differences in the number of core SNPs identified in the 30 emetic group III isolates using different variant calling pipelines, the pipelines that used assembled genomes as input (kSNP3 and Parsnp) produced higher numbers of core SNPs than their counterparts that relied on short Illumina reads. Additionally, when used to query core SNPs in 55 emetic group III ST 26 *B. cereus* group genomes, both kSNP3 and Parsnp produced core SNPs that yielded topologically similar phylogenies. kSNP3 employs a reference-free *k-*mer based SNP calling approach (Gardner and Hall, 2013; Gardner et al., 2015), while Parsnp uses a reference-based core genome alignment approach (Treangen et al., 2014), and both are useful for calling variants in large data sets. These approaches are also valuable when reads are not available for SNP calling (Olson et al., 2015), as demonstrated here by the comparison of outbreak genomes with publicly available genomes: core SNPs obtained using both kSNP3 and Parsnp were able to consistently produce phylogenies in which the 30 emetic isolates from this outbreak formed a well-supported clade among all emetic group III ST 26 *B. cereus* group genomes. However, kSNP3 has been shown to lack specificity relative to other pipelines (i.e., CFSAN, LYVE-SET) when differentiating outbreak isolates from non-outbreak isolates for *L. monocytogenes, E. coli*, and *S. enterica* (Katz et al., 2017). Here, the CFSAN and LYVE-SET pipelines identified similar SNPs that produced highly congruent phylogenies. This is unsurprising, considering that both the CFSAN and LYVE-SET pipelines were designed specifically for identifying SNPs in closely related strains from outbreaks (Katz et al., 2017), and both employ the most stringent filtering criteria of all pipelines tested here.

### As WGS Becomes Routinely Integrated Into Food Safety, Clinical, and Epidemiological Realms, It Is Likely That the Number of Illnesses Attributed to *B. cereus* Will Increase

Here, we offer the first description of a foodborne outbreak caused by *B. cereus* group species to be characterized using WGS, and we provide a glimpse into the genomic variation one might expect within an emetic group III *B. cereus* outbreak using several different variant calling pipelines. However, our ability to query emetic group III genomes outside of this outbreak is limited by the lack of publicly available genomic data and metadata from emetic isolates. Of the 2,156 *B. cereus* group genomes available in NCBI's RefSeq database as of March 2018, only 29 were from group III and possessed the *cesABCD* operon, 25 of which belonged to MLST ST 26. While not ideal, this is an improvement, as there were only 19 emetic group III genomes available in NCBI's Genbank database in April 2017 (Carroll et al., 2017). As more *B. cereus* group WGS data—particularly, data from emetic *B. cereus* group isolates—become publicly available, more outbreaks and clusters are likely to be resolved in tandem, a phenomenon that has been observed for *L. monocytogenes* (Jackson et al., 2016). Additionally, variant calling and cluster/outbreak detection methods for characterizing *B. cereus* group isolates from foodborne outbreaks can be further refined and optimized as more WGS, metadata and epidemiological data become available for clinical and non-clinical isolates.

## Author Contributions

LC performed computational analyses. MM, LM, ND, and JC performed microbiological experiments. DN provided and interpreted epidemiological data. MW and JK conceived the study. LC, MW, and JK co-wrote the manuscript.

### Conflict of Interest Statement

The authors declare that the research was conducted in the absence of any commercial or financial relationships that could be construed as a potential conflict of interest.

## References

[B1] Allard M. W. Bell R. Ferreira C. M. Gonzalez-Escalona N. Hoffmann M. Muruvanda T.. (2017). Genomics of foodborne pathogens for microbial food safety. Curr. Opin. Biotechnol. 49, 224–229. 10.1016/j.copbio.2017.11.002 29169072

[B2] Anderson M. J. Walsh D. C. I. (2013). PERMANOVA, ANOSIM, and the mantel test in the face of heterogeneous dispersions: what null hypothesis are you testing? Ecol. Monographs 83, 557–574. 10.1890/12-2010.1

[B3] Andersson M. A. Jääskeläinen E. L. Shaheen R. Pirhonen T. Wijnands L. M. Salkinoja-Salonen M. S. (2004). Sperm bioassay for rapid detection of cereulide-producing *Bacillus cereus* in food and related environments. Int. J. Food Microbiol. 94, 175–183. 10.1016/j.ijfoodmicro.2004.01.018 15193804

[B4] Ashton P. Nair S. Peters T. Tewolde R. Day M. Doumith M. (2015). Revolutionising public health reference microbiology using whole genome sequencing: *Salmonella* as an exemplar. *bioRxiv* 033225. 10.1101/033225

[B5] Bankevich A. Nurk S. Antipov D. Gurevich A. A. Dvorkin M. Kulikov A. S.. (2012). SPAdes: a new genome assembly algorithm and its applications to single-cell sequencing. J. Comput. Biol. 19, 455–477. 10.1089/cmb.2012.0021 22506599 PMC3342519

[B6] Bennett S. D. Walsh K. A. Gould L. H. (2013). Foodborne disease outbreaks caused by *Bacillus cereus, Clostridium perfringens*, and *Staphylococcus aureus*–United States, 1998–2008. Clin. Infect. Dis. 57, 425–433. 10.1093/cid/cit244 23592829 PMC11334977

[B7] Bolger A. M. Lohse M. Usadel B. (2014). Trimmomatic: a flexible trimmer for illumina sequence data. Bioinformatics 30, 2114–2120. 10.1093/bioinformatics/btu170 24695404 PMC4103590

[B8] Bruen T. C. Philippe H. Bryant D. (2006). A simple and robust statistical test for detecting the presence of recombination. Genetics 172, 2665–2681. 10.1534/genetics.105.048975 16489234 PMC1456386

[B9] Carroll L. M. Kovac J. Miller R. A. Wiedmann M. (2017). Rapid, high-throughput identification of anthrax-causing and emetic *Bacillus cereus* group genome assemblies using BTyper, a computational tool for virulence-based classification of *Bacillus cereus* group isolates using nucleotide sequencing data. Appl. Environ. Microbiol. 83:e01096-17 10.1128/AEM.01096-17 PMC5561296 28625989

[B10] Castiaux V. Liu X. Delbrassinne L. Mahillon J. (2015). Is cytotoxin K from *Bacillus cereus* a bona fide enterotoxin? Int. J. Food Microbiol. 211, 79–85. 10.1016/j.ijfoodmicro.2015.06.020 26186121

[B11] Ceuppens S. Boon N. Uyttendaele M. (2013). Diversity of *Bacillus cereus* group strains is reflected in their broad range of pathogenicity and diverse ecological lifestyles. FEMS Microbiol. Ecol. 84, 433–450. 10.1111/1574-6941.12110 23488744

[B12] Chen Y. Luo Y. Curry P. Timme R. Melka D. Doyle M.. (2017a). Assessing the genome level diversity of *Listeria monocytogenes* from contaminated ice cream and environmental samples linked to a listeriosis outbreak in the United States. PLoS ONE 12:e0171389. 10.1371/journal.pone.0171389 28166293 PMC5293252

[B13] Chen Y. Luo Y. Pettengill J. Timme R. Melka D. Doyle M.. (2017b). Singleton sequence type 382, an emerging clonal group of *Listeria monocytogenes* associated with three multistate outbreaks linked to contaminated stone fruit, caramel apples, and leafy green salad. J. Clin. Microbiol. 55, 931–941. 10.1128/JCM.02140-16 28053218 PMC5328462

[B14] Clarke K. R. (1993). Non-parametric multivariate analysis of changes in community structure. Aust. J. Ecol. 18, 117–143. 10.1111/j.1442-9993.1993.tb00438.x

[B15] Croucher N. J. Page A. J. Connor T. R. Delaney A. J. Keane J. A. Bentley S. D.. (2015). Rapid phylogenetic analysis of large samples of recombinant bacterial whole genome sequences using gubbins. Nucleic Acids Res. 43:e15. 10.1093/nar/gku1196 25414349 PMC4330336

[B16] Danecek P. Auton A. Abecasis G. Albers C. A. Banks E. DePristo M. A.. (2011). The variant call format and VCFtools. Bioinformatics 27, 2156–2158. 10.1093/bioinformatics/btr330 21653522 PMC3137218

[B17] Davis S. Pettengill J. B. Luo Y. Payne J. Shpuntoff A. Rand H. (2015). CFSAN SNP Pipeline: an automated method for constructing SNP matrices from next-generation sequence data. PeerJ Comput. Sci. 1:e20 10.7717/peerj-cs.20

[B18] de Jonge E. (2016). Docopt: Command-Line Interface Specification Language. R package version 0.4.5. Available online at: https://CRAN.R-project.org/package=docopt

[B19] Doll V. M. Ehling-Schulz M. Vogelmann R. (2013). Concerted action of sphingomyelinase and non-hemolytic enterotoxin in pathogenic *Bacillus cereus*. PLoS ONE 8:e61404. 10.1371/journal.pone.0061404 23613846 PMC3628865

[B20] Ehling-Schulz M. Frenzel E. Gohar M. (2015). Food-bacteria interplay: pathometabolism of emetic *Bacillus cereus*. Front. Microbiol. 6:704. 10.3389/fmicb.2015.00704 26236290 PMC4500953

[B21] Ehling-Schulz M. Fricker M. Scherer S. (2004). *Bacillus cereus*, the causative agent of an emetic type of food-borne illness. Mol. Nutr. Food Res. 48, 479–487. 10.1002/mnfr.200400055 15538709

[B22] Ehling-Schulz M. Svensson B. Guinebretiere M. H. Lindbäck T. Andersson M. Schulz A.. (2005). Emetic toxin formation of *Bacillus cereus* is restricted to a single evolutionary lineage of closely related strains. Microbiology 151(Pt 1), 183–197. 10.1099/mic.0.27607-0 15632437

[B23] Fisichella M. Dabboue H. Bhattacharyya S. Saboungi M. L. Salvetat J. P. Hevor T.. (2009). Mesoporous silica nanoparticles enhance MTT formazan exocytosis in HeLa cells and astrocytes. Toxicol. In Vitro 23, 697–703. 10.1016/j.tiv.2009.02.007 19254755

[B24] Gardner S. N. Hall B. G. (2013). When whole-genome alignments just won't work: kSNP v2 software for alignment-free SNP discovery and phylogenetics of hundreds of microbial genomes. PLoS ONE 8:e81760. 10.1371/journal.pone.0081760 24349125 PMC3857212

[B25] Gardner S. N. Slezak T. Hall B. G. (2015). kSNP3.0: SNP detection and phylogenetic analysis of genomes without genome alignment or reference genome. Bioinformatics 31, 2877–2878. 10.1093/bioinformatics/btv271 25913206

[B26] Garrison E. Marth G. (2012). Haplotype-based variant detection from short-read sequencing. arXiv preprint arXiv:1207.3907 [q-bio.GN].

[B27] Ghosh A. C. (1978). Prevalence of *Bacillus cereus* in the faeces of healthy adults. J. Hyg. 80, 233–236. 10.1017/S0022172400053572 416141 PMC2130004

[B28] Glasset B. Herbin S. Guillier L. Cadel-Six S. Vignaud M. L. Grout J.. (2016). *Bacillus cereus*-induced food-borne outbreaks in France, 2007 to 2014: epidemiology and genetic characterisation. Euro. Surveill. 21:30413. 10.2807/1560-7917.ES.2016.21.48.30413 27934583 PMC5388111

[B29] Grad Y. H. Lipsitch M. Feldgarden M. Arachchi H. M. Cerqueira G. C. Fitzgerald M.. (2012). Genomic epidemiology of the *Escherichia coli* O104:H4 outbreaks in Europe, 2011. Proc. Natl. Acad. Sci. U.S.A. 109, 3065–3070. 10.1073/pnas.1121491109 22315421 PMC3286951

[B30] Granum P. E. Lund T. (1997). *Bacillus cereus* and its food poisoning toxins. FEMS Microbiol. Lett. 157, 223–228. 10.1111/j.1574-6968.1997.tb12776.x 9435100

[B31] Guangchuang Y. Smith D. K. Huachen Z. Yi G. Tsan-Yuk L.T. (2017). ggtree: an r package for visualization and annotation of phylogenetic trees with their covariates and other associated data. Methods Ecol. Evolut. 8, 28–36. 10.1111/2041-210X.12628

[B32] Guinebretière M. H. Auger S. Galleron N. Contzen M. De Sarrau B. De Buyser M. L.. (2013). *Bacillus cytotoxicus* sp. nov. is a novel thermotolerant species of the *Bacillus cereus* group occasionally associated with food poisoning. Int. J. Syst. Evol. Microbiol. 63(Pt 1), 31–40. 10.1099/ijs.0.030627-0 22328607

[B33] Guinebretière M. H. Thompson F. L. Sorokin A. Normand P. Dawyndt P. Ehling-Schulz M.. (2008). Ecological diversification in the *Bacillus cereus* group. Environ. Microbiol. 10, 851–865. 10.1111/j.1462-2920.2007.01495.x 18036180

[B34] Guinebretiere M. H. Velge P. Couvert O. Carlin F. Debuyser M. L. Nguyen-The C. (2010). Ability of *Bacillus cereus* group strains to cause food poisoning varies according to phylogenetic affiliation (groups I to VII) rather than species affiliation. J. Clin. Microbiol. 48, 3388–3391. 10.1128/JCM.00921-10 20660215 PMC2937725

[B35] Gymoese P. Sørensen G. Litrup E. Olsen J. E. Nielsen E. M. Torpdahl M. (2017). Investigation of outbreaks of *Salmonella enterica* serovar typhimurium and its monophasic variants using whole-genome sequencing, Denmark. Emerg. Infect. Dis. 23, 1631–1639. 10.3201/eid2310.161248 28930002 PMC5621559

[B36] Hackathon R. Bolker B. Butler M. Cowan P. de Vienne D. Eddelbuettel D. (2017). Phylobase: Base Package for Phylogenetic Structures and Comparative Data. R package version 0.8.4. Available online at: https://CRAN.R-project.org/package=phylobase

[B37] Heibl C. (2008). PHYLOCH: R Language Tree Plotting Tools and Interfaces To Diverse Phylogenetic Software Packages. Avaliable online at: http://www.christophheibl.de/Rpackages.html.

[B38] Hoffmann M. Luo Y. Monday S. R. Gonzalez-Escalona N. Ottesen A. R. Muruvanda T.. (2016). Tracing origins of the *Salmonella* bareilly strain causing a food-borne outbreak in the United States. J. Infect. Dis. 213, 502–508. 10.1093/infdis/jiv297 25995194

[B39] Holmes A. Allison L. Ward M. Dallman T. J. Clark R. Fawkes A.. (2015). Utility of whole-genome sequencing of *Escherichia coli* O157 for outbreak detection and epidemiological surveillance. J. Clin. Microbiol. 53, 3565–3573. 10.1128/JCM.01066-15 26354815 PMC4609728

[B40] Hwang S. Kim E. Lee I. Marcotte E. M. (2015). Systematic comparison of variant calling pipelines using gold standard personal exome variants. Sci. Rep. 5:17875. 10.1038/srep17875 26639839 PMC4671096

[B41] Ivy R. A. Ranieri M. L. Martin N. H. den Bakker H. C. Xavier B. M. Wiedmann M.. (2012). Identification and characterization of psychrotolerant sporeformers associated with fluid milk production and processing. Appl. Environ. Microbiol. 78, 1853–1864. 10.1128/AEM.06536-11 22247129 PMC3298126

[B42] Jackson B. R. Tarr C. Strain E. Jackson K. A. Conrad A. Carleton H.. (2016). Implementation of nationwide real-time whole-genome sequencing to enhance listeriosis outbreak detection and investigation. Clin. Infect. Dis. 63, 380–386. 10.1093/cid/ciw242 27090985 PMC4946012

[B43] Jain C. Rodriguez-R L. M. Phillippy A. M. Konstantinidis K. T. Aluru S. (2018). High throughput ANI analysis of 90K prokaryotic genomes reveals clear species boundaries. Nat. Commun. 9:5114. 10.1038/s41467-018-07641-9 30504855 PMC6269478

[B44] Jakobsen I. B. Easteal S. (1996). A program for calculating and displaying compatibility matrices as an aid in determining reticulate evolution in molecular sequences. Comput. Appl. Biosci. 12, 291–295. 10.1093/bioinformatics/12.4.291 8902355

[B45] Jiménez G. Urdiain M. Cifuentes A. López-López A. Blanch A. R. Tamames J.. (2013). Description of *Bacillus toyonensis* sp. nov., a novel species of the Bacillus cereus group, and pairwise genome comparisons of the species of the group by means of ANI calculations. Syst. Appl. Microbiol. 36, 383–391. 10.1016/j.syapm.2013.04.008 23791203

[B46] Joensen K. G. Scheutz F. Lund O. Hasman H. Kaas R. S. Nielsen E. M.. (2014). Real-time whole-genome sequencing for routine typing, surveillance, and outbreak detection of verotoxigenic *Escherichia coli*. J. Clin. Microbiol. 52, 1501–1510. 10.1128/JCM.03617-13 24574290 PMC3993690

[B47] Jombart T. Kendall M. Almagro-Garcia J. Colijn C. (2017). Treespace: statistical exploration of landscapes of phylogenetic trees. Mol. Ecol. Resour. 17, 1385–1392. 10.1111/1755-0998.12676 28374552 PMC5724650

[B48] Kabir M. S. Hsieh Y. H. Simpson S. Kerdahi K. Sulaiman I. M. (2017). Evaluation of two standard and two chromogenic selective media for optimal growth and enumeration of isolates of 16 unique *Bacillus* species. J. Food Prot. 80:952–962. 10.4315/0362-028X.JFP-16-441 28467187

[B49] Katz L. S. Griswold T. Williams-Newkirk A. J. Wagner D. Petkau A. Sieffert C.. (2017). A comparative analysis of the lyve-set phylogenomics pipeline for genomic epidemiology of foodborne pathogens. Front. Microbiol. 8:375. 10.3389/fmicb.2017.00375 28348549 PMC5346554

[B50] Kendall M. Colijn C. (2015). A tree metric using structure and length to capture distinct phylogenetic signals. arXiv:1507.05211v3 [q-bio.PE].

[B51] Kovac J. Bakker H. D. Carroll L. M. Wiedmann M. (2017). Precision food safety: a systems approach to food safety facilitated by genomics tools. TrAC Trends Analy. Chem. 96(Suppl. C), 52–61. 10.1016/j.trac.2017.06.001

[B52] Kovac J. Miller R. A. Carroll L. M. Kent D. J. Jian J. Beno S. M.. (2016). Production of hemolysin BL by *Bacillus cereus* group isolates of dairy origin is associated with whole-genome phylogenetic clade. BMC Genomics 17:581. 10.1186/s12864-016-2883-z 27507015 PMC4979109

[B53] Kwong J. C. Mercoulia K. Tomita T. Easton M. Li H. Y. Bulach D. M.. (2016). Prospective whole-genome sequencing enhances national surveillance of *Listeria monocytogenes*. J. Clin. Microbiol. 54, 333–342. 10.1128/JCM.02344-15 26607978 PMC4733179

[B54] Lewis P. O. (2001). A likelihood approach to estimating phylogeny from discrete morphological character data. Syst. Biol. 50, 913–925. 10.1080/106351501753462876 12116640

[B55] Li H. (2013). Aligning sequence reads, clone sequences and assembly contigs with BWA-MEM. arXiv:1303.3997v1 [q-bio.GN].

[B56] Li H. Durbin R. (2010). Fast and accurate long-read alignment with burrows-wheeler transform. Bioinformatics 26, 589–595. 10.1093/bioinformatics/btp698 20080505 PMC2828108

[B57] Li H. Handsaker B. Wysoker A. Fennell T. Ruan J. Homer N.. (2009). The sequence alignment/map format and SAMtools. Bioinformatics 25, 2078–2079. 10.1093/bioinformatics/btp352 19505943 PMC2723002

[B58] Liu Y. Du J. Lai Q. Zeng R. Ye D. Xu J.. (2017). Proposal of nine novel species of the *Bacillus cereus* group. Int. J. Syst. Evol. Microbiol. 67, 2499–2508. 10.1099/ijsem.0.001821 28792367

[B59] Lotte R. Hérissé A. L. Berrouane Y. Lotte L. Casagrande F. Landraud L.. (2017). Virulence analysis of *bacillus cereus* isolated after death of preterm neonates, nice, France, 2013. Emerg. Infect. Dis. 23, 845–848. 10.3201/eid2305.161788 28418291 PMC5403044

[B60] Mair-Jenkins J. Borges-Stewart R. Harbour C. Cox-Rogers J. Dallman T. Ashton P.. (2017). Investigation using whole genome sequencing of a prolonged restaurant outbreak of *Salmonella* Typhimurium linked to the building drainage system, England, February 2015 to March 2016. Euro. Surveill. 22:17-00037. 10.2807/1560-7917.ES.2017.22.49.17-00037 29233257 PMC5727591

[B61] McCloskey R. M. Poon A. F. Y. (2017). A model-based clustering method to detect infectious disease transmission outbreaks from sequence variation. PLoS Comput. Biol. 13:e1005868. 10.1371/journal.pcbi.1005868 29131825 PMC5703573

[B62] Messelhäusser U. Frenzel E. Blöchinger C. Zucker R. Kämpf P. Ehling-Schulz M. (2014). Emetic *Bacillus cereus* are more volatile than thought: recent foodborne outbreaks and prevalence studies in Bavaria (2007–2013). Biomed. Res. Int. 2014:465603. 10.1155/2014/465603 24895578 PMC4033357

[B63] Miller R. A. Beno S. M. Kent D. J. Carroll L. M. Martin N. H. Boor K. J.. (2016). Bacillus wiedmannii sp. nov., a psychrotolerant and cytotoxic bacillus cereus group species isolated from dairy foods and dairy environments. Int. J. Syst. Evol. Microbiol. 66, 4744–4753. 10.1099/ijsem.0.001421 27520992 PMC5381181

[B64] Miller R. A. Jian J. Beno S. M. Wiedmann M. Kovac J. (2018). Intraclade variability in toxin production and cytotoxicity of *bacillus cereus* group type strains and dairy-associated isolates. Appl. Environ. Microbiol. 84:e02479–17. 10.1128/AEM.02479-17 29330180 PMC5835744

[B65] Moran-Gilad J. (2017). Whole genome sequencing (WGS) for food-borne pathogen surveillance and control–taking the pulse. Euro. Surveill. 22:30547. 10.2807/1560-7917.ES.2017.22.23.30547 28661389 PMC5479979

[B66] Morgulis A. Gertz E. M. Schäffer A. A. Agarwala R. (2006). A fast and symmetric DUST implementation to mask low-complexity DNA sequences. J. Comput. Biol. 13, 1028–1040. 10.1089/cmb.2006.13.1028 16796549

[B67] Moura A. Tourdjman M. Leclercq A. Hamelin E. Laurent E. Fredriksen N.. (2017). Real-time whole-genome sequencing for surveillance of *Listeria monocytogenes*, France. Emerg. Infect. Dis. 23, 1462–1470. 10.3201/eid2309.170336 28643628 PMC5572858

[B68] Naranjo M. Denayer S. Botteldoorn N. Delbrassinne L. Veys J. Waegenaere J.. (2011). Sudden death of a young adult associated with *Bacillus cereus* food poisoning. J. Clin. Microbiol. 49, 4379–4381. 10.1128/JCM.05129-11 22012017 PMC3232990

[B69] Oksanen J. Blanchet F. G. Friendly M. Kindt R. Legendre P. McGlinn D. (2018). Vegan: Community Ecology Package. R package version 2.5-2. Available online at: https://CRAN.R-project.org/package=vegan

[B70] Olson N. D. Lund S. P. Colman R. E. Foster J. T. Sahl J. W. Schupp J. M.. (2015). Best practices for evaluating single nucleotide variant calling methods for microbial genomics. Front. Genet. 6:235. 10.3389/fgene.2015.00235 26217378 PMC4493402

[B71] Paradis E. Claude J. Strimmer K. (2004). APE: analyses of phylogenetics and evolution in R language. Bioinformatics 20, 289–290. 10.1093/bioinformatics/btg412 14734327

[B72] Pightling A. W. Petronella N. Pagotto F. (2014). Choice of reference sequence and assembler for alignment of *Listeria monocytogenes* short-read sequence data greatly influences rates of error in SNP analyses. PLoS ONE 9:e104579. 10.1371/journal.pone.0104579 25144537 PMC4140716

[B73] Pightling A. W. Petronella N. Pagotto F. (2015). Choice of reference-guided sequence assembler and SNP caller for analysis of *Listeria monocytogenes* short-read sequence data greatly influences rates of error. BMC Res. Notes 8:748. 10.1186/s13104-015-1689-4 26643440 PMC4672502

[B74] Pruitt K. D. Tatusova T. Maglott D. R. (2007). NCBI reference sequences (RefSeq): a curated non-redundant sequence database of genomes, transcripts and proteins. Nucleic Acids Res. 35, D61–D65. 10.1093/nar/gkl,842 17130148 PMC1716718

[B75] R Core Team. (2018). R: A language and Environment for Statistical Computing. Vienna: R Foundation for Statistical Computing. Available online at: https://www.R-project.org,

[B76] Revell L. J. (2012). phytools: an R package for phylogenetic comparative biology (and other things). Methods Ecol. Evol. 3, 217–223. 10.1111/j.2041-210X.2011.00169.x

[B77] Rusconi B. Sanjar F. Koenig S. S. Mammel M. K. Tarr P. I. Eppinger M. (2016). Whole genome sequencing for genomics-guided investigations of *Escherichia coli* O157:H7 outbreaks. Front. Microbiol. 7:985. 10.3389/fmicb.2016.00985 27446025 PMC4928038

[B78] Sanaei-Zadeh H. (2012). Can *Bacillus cereus* food poisoning cause sudden death? J. Clin. Microbiol. 50, 3816–3817. 10.1128/JCM.00059-12 23066123 PMC3486252

[B79] Sandmann S. de Graaf A. O. Karimi M. van der Reijden B. A. Hellström-Lindberg E. Jansen J. H.. (2017). Evaluating variant calling tools for non-matched next-generation sequencing data. Sci. Rep. 7:43169. 10.1038/srep43169 28233799 PMC5324109

[B80] Scallan E. Hoekstra R. M. Angulo F. J. Tauxe R. V. Widdowson M. A. Roy S. L.. (2011). Foodborne illness acquired in the United States–major pathogens. Emerg. Infect. Dis. 17, 7–15. 10.3201/eid1701.P11101 21192848 PMC3375761

[B81] Schliep K. P. (2011). Phangorn: phylogenetic analysis in R. Bioinformatics 27, 592–593. 10.1093/bioinformatics/btq706 21169378 PMC3035803

[B82] Schoeni J. L. Wong A. C. (2005). *Bacillus cereus* food poisoning and its toxins. J. Food Prot. 68, 636–648. 10.4315/0362-028X-68.3.636 15771198

[B83] Smith J. M. (1992). Analyzing the mosaic structure of genes. J. Mol. Evol. 34, 126–129. 10.1007/BF00182389 1556748

[B84] Stamatakis A. (2014). RAxML version 8: a tool for phylogenetic analysis and post-analysis of large phylogenies. Bioinformatics 30, 1312–1313. 10.1093/bioinformatics/btu033 24451623 PMC3998144

[B85] Stenfors Arnesen L. P. Fagerlund A. Granum P. E. (2008). From soil to gut: *Bacillus cereus* and its food poisoning toxins. FEMS Microbiol. Rev. 32, 579–606. 10.1111/j.1574-6976.2008.00112.x 18422617

[B86] Taboada E. N. Graham M. R. Carriço J. A. Van Domselaar G. (2017). Food safety in the age of next generation sequencing, bioinformatics, and open data access. Front. Microbiol. 8:909. 10.3389/fmicb.2017.00909 28588568 PMC5440521

[B87] Tallent S. M. Kotewicz K. M. Strain E. A. Bennett R. W. (2012a). Efficient isolation and identification of *Bacillus cereus* group. J. AOAC Int. 95, 446–451. 10.5740/jaoacint.11-251 22649932

[B88] Tallent S. M. Rhodehamel E. J. Harmon S. M. Bennett R. W. (2012b). Chapter 14: Bacillus cereus, in Bacteriological Analytical Manual (Silver Spring, MD: U.S. Food and Drug Administration). Available online at: https://www.fda.gov/Food/FoodScienceResearch/LaboratoryMethods/ucm070875.htm

[B89] Taylor A. J. Lappi V. Wolfgang W. J. Lapierre P. Palumbo M. J. Medus C.. (2015). Characterization of foodborne outbreaks of *Salmonella enterica* serovar enteritidis with whole-genome sequencing single nucleotide polymorphism-based analysis for surveillance and outbreak detection. J. Clin. Microbiol. 53, 3334–3340. 10.1128/JCM.01280-15 26269623 PMC4572550

[B90] Treangen T. J. Ondov B. D. Koren S. Phillippy A. M. (2014). The Harvest suite for rapid core-genome alignment and visualization of thousands of intraspecific microbial genomes. Genome. Biol. 15:524. 10.1186/s13059-014-0524-x 25410596 PMC4262987

[B91] Turnbull P. C. Kramer J. M. (1985). Intestinal carriage of *Bacillus cereus*: faecal isolation studies in three population groups. J. Hyg. (Lond). 95, 629–638. 10.1017/S0022172400060733 3937856 PMC2129566

[B92] Usongo V. Berry C. Yousfi K. Doualla-Bell F. Labbe G. Johnson R.. (2018). Impact of the choice of reference genome on the ability of the core genome SNV methodology to distinguish strains of *Salmonella enterica* serovar Heidelberg. PLoS ONE 13:e0192233. 10.1371/journal.pone.0192233 29401524 PMC5798827

[B93] Vangay P. Fugett E. B. Sun Q. Wiedmann M. (2013). Food microbe tracker: a web-based tool for storage and comparison of food-associated microbes. J. Food. Prot. 76, 283–294. 10.4315/0362-028X.JFP-12-276 23433376

[B94] Walker T. M. Merker M. Knoblauch A. M. Helbling P. Schoch O. D. van der Werf M. J.. (2018). A cluster of multidrug-resistant *Mycobacterium tuberculosis* among patients arriving in Europe from the Horn of Africa: a molecular epidemiological study. Lancet Infect. Dis. 18, 431–440. 10.1016/S1473-3099(18)30004-5 29326013 PMC5864516

[B95] Wickham H. (2018). Stringr: Simple, Consistent Wrappers for Common String Operations. R package version 1.3.1. Available online at: https://CRAN.R-project.org/package=stringr

[B96] Ye J. McGinnis S. Madden T. L. (2006). BLAST: improvements for better sequence analysis. Nucleic Acids Res. 34, W6–W9. 10.1093/nar/gkl164 16845079 PMC1538791

