# Supplementary material for: Characterization of Emetic and Diarrheal Bacillus cereus Strains From a 2016 Foodborne Outbreak Using Whole-Genome Sequencing: Addressing the Microbiological, Epidemiological, and Bioinformatic Challenges
Source: Front Microbiol. 2019 Feb 12;10:144. doi: 10.3389/fmicb.2019.00144 (PMC6379260; doi:10.3389/fmicb.2019.00144)
Supplement: Supplementary file 1 [file Table_1.DOCX]

**Supplementary Table S1.** *Bacillus cereus* isolates sequenced in conjunction with this outbreak.

| **Study ID** | **FMT ID^a^** | **SRA Accession^b^** | **Biosample**  **Accession** | **Per-Base Average Coverage** | **# of**  **Contigs** | **Contig**  **N50** | **Source,**  **General** | **Source,**  **Specific** | **Isolation**  **Date** |
| --- | --- | --- | --- | --- | --- | --- | --- | --- | --- |
| FOOD_10_18_16_LFTOV_NA_R9-6400 | FSL R9-6400 | SRR6825047 | SAMN08685572 | 73.1065 | 195 | 115822 | Food | Leftovers | 10/18/16 |
| FOOD_10_18_16_LFTOV_NA_R9-6401 | FSL R9-6401 | SRR6825048 | SAMN08685573 | 44.9643 | 248 | 54551 | Food | Leftovers | 10/18/16 |
| FOOD_10_18_16_LFTOV_NA_R9-6402 | FSL R9-6402 | SRR6825049 | SAMN08685574 | 74.0628 | 273 | 64233 | Food | Leftovers | 10/18/16 |
| FOOD_10_19_16_RSNT1_1B_R9-6388 | FSL R9-6388 | SRR6825034 | SAMN08685563 | 78.5668 | 535 | 94860 | Food | Restaurant 1 | 10/19/16 |
| FOOD_10_19_16_RSNT1_1B_R9-6389 | FSL R9-6389 | SRR6827986 | SAMN08688985 | 66.5878 | 295 | 55012 | Food | Restaurant 1 | 10/19/16 |
| FOOD_10_19_16_RSNT1_1B_R9-6390 | FSL R9-6390 | SRR6825035 | SAMN08685564 | 66.6441 | 434 | 105997 | Food | Restaurant 1 | 10/19/16 |
| FOOD_10_19_16_RSNT1_1B_R9-6391 | FSL R9-6391 | SRR6825036 | SAMN08685565 | 93.6519 | 407 | 74128 | Food | Restaurant 1 | 10/19/16 |
| FOOD_10_19_16_RSNT1_2A_R9-6386 | FSL R9-6386 | SRR6825032 | SAMN08685561 | 70.4816 | 493 | 40729 | Food | Restaurant 1 | 10/19/16 |
| FOOD_10_19_16_RSNT1_2A_R9-6387 | FSL R9-6387 | SRR6825033 | SAMN08685562 | 74.2635 | 462 | 77833 | Food | Restaurant 1 | 10/19/16 |
| FOOD_10_19_16_RSNT1_2H_R9-6392 | FSL R9-6392 | SRR6825037 | SAMN08685566 | 88.1972 | 394 | 64490 | Food | Restaurant 1 | 10/19/16 |
| FOOD_10_19_16_RSNT1_2H_R9-6393 | FSL R9-6393 | SRR6825038 | SAMN08685567 | 124.493 | 144 | 90157 | Food | Restaurant 1 | 10/19/16 |
| FOOD_10_19_16_RSNT1_2H_R9-6394 | FSL R9-6394 | SRR6825039 | SAMN08685568 | 67.6895 | 385 | 95754 | Food | Restaurant 1 | 10/19/16 |
| FOOD_10_19_16_RSNT1_2H_R9-6395 | FSL R9-6395 | SRR6827985 | SAMN08688986 | 84.685 | 990 | 43107 | Food | Restaurant 1 | 10/19/16 |
| FOOD_10_19_16_RSNT1_2H_R9-6396 | FSL R9-6396 | SRR6825040 | SAMN08685569 | 74.4779 | 344 | 98339 | Food | Restaurant 1 | 10/19/16 |
| FOOD_10_19_16_RSNT2_2A_R9-6397 | FSL R9-6397 | SRR6825041 | SAMN08685570 | 90.0117 | 623 | 106372 | Food | Restaurant 2 | 10/19/16 |
| FOOD_10_19_16_RSNT2_2A_R9-6398 | FSL R9-6398 | SRR6825046 | SAMN08685571 | 87.8401 | 480 | 81304 | Food | Restaurant 2 | 10/19/16 |
| FOOD_10_19_16_RSNT2_2A_R9-6399 | FSL R9-6399 | SRR6827984 | SAMN08688987 | 117.942 | 947 | 56443 | Food | Restaurant 2 | 10/19/16 |
| FOOD_10_19_16_RSNT3_1E_R9-6406 | FSL R9-6406 | SRR6827983 | SAMN08688988 | 229.125 | 148 | 251432 | Food | Restaurant 3 | 10/19/16 |
| FOOD_10_19_16_RSNT3_1E_R9-6407 | FSL R9-6407 | SRR6825045 | SAMN08685578 | 67.0677 | 207 | 105938 | Food | Restaurant 3 | 10/19/16 |
| FOOD_10_19_16_RSNT3_2A_R9-6403 | FSL R9-6403 | SRR6825042 | SAMN08685575 | 67.9815 | 336 | 90693 | Food | Restaurant 3 | 10/19/16 |
| FOOD_10_19_16_RSNT3_2A_R9-6404 | FSL R9-6404 | SRR6825043 | SAMN08685576 | 76.355 | 261 | 105269 | Food | Restaurant 3 | 10/19/16 |
| FOOD_10_19_16_RSNT3_2A_R9-6405 | FSL R9-6405 | SRR6825044 | SAMN08685577 | 76.0559 | 313 | 75288 | Food | Restaurant 3 | 10/19/16 |
| FOOD_10_19_16_RSNT4_2B_R9-6408 | FSL R9-6408 | SRR6825050 | SAMN08685579 | 62.0725 | 232 | 115539 | Food | Restaurant 4 | 10/19/16 |
| FOOD_10_19_16_RSNT4_2B_R9-6409 | FSL R9-6409 | SRR6825051 | SAMN08685580 | 82.283 | 282 | 82242 | Food | Restaurant 4 | 10/19/16 |
| FOOD_10_19_16_RSNT5_1C_R9-6410 | FSL R9-6410 | SRR6827982 | SAMN08688989 | 213.004 | 306 | 91817 | Food | Restaurant 5 | 10/19/16 |
| FOOD_10_19_16_RSNT5_1C_R9-6411 | FSL R9-6411 | SRR6825029 | SAMN08685581 | 90.3287 | 465 | 59098 | Food | Restaurant 5 | 10/19/16 |
| HUMN_10_18_16_FECAL_NA_R9-6384 | FSL R9-6384 | SRR6827987 | SAMN08688984 | 86.8609 | 1204 | 12251 | Human | Fecal | 10/18/16 |
| HUMN_10_18_16_FECAL_NA_R9-6385 | FSL R9-6385 | SRR6825031 | SAMN08685583 | 76.5514 | 406 | 51112 | Human | Fecal | 10/18/16 |
| HUMN_10_18_16_FECAL_NA_R9-6412 | FSL R9-6412 | SRR6825030 | SAMN08685584 | 40.0285 | 312 | 39332 | Human | Fecal | 10/18/16 |
| HUMN_10_19_16_FECAL_NA_R9-6381 | FSL R9-6381 | SRR6827989 | SAMN08688982 | 83.1124 | 1128 | 12271 | Human | Fecal | 10/19/16 |
| HUMN_10_19_16_FECAL_NA_R9-6382 | FSL R9-6382 | SRR6827988 | SAMN08688983 | 214.56 | 283 | 69159 | Human | Fecal | 10/19/16 |
| HUMN_10_19_16_FECAL_NA_R9-6383 | FSL R9-6383 | SRR6825028 | SAMN08685582 | 68.3741 | 397 | 49140 | Human | Fecal | 10/19/16 |
| HUMN_10_26_16_FECAL_NA_R9-6413 | FSL R9-6413 | SRR6827981 | SAMN08688990 | 54.7695 | 913 | 22415 | Human | Fecal | 10/26/16 |

^a^FMT ID; Food Microbe Tracker identification number.

^b^SRA; Sequence Read Archive
