# Supplementary material for: Characterization of Emetic and Diarrheal Bacillus cereus Strains From a 2016 Foodborne Outbreak Using Whole-Genome Sequencing: Addressing the Microbiological, Epidemiological, and Bioinformatic Challenges
Source: Front Microbiol. 2019 Feb 12;10:144. doi: 10.3389/fmicb.2019.00144 (PMC6379260; doi:10.3389/fmicb.2019.00144)
Supplement: Supplementary file 2 [file Table_2.DOCX]

**Supplementary Table S2.** Genomes for 18 currently-recognized *B. cereus* group species used in this study.

| **Species^a^** | **Strain** | **RefSeq Accession** |
| --- | --- | --- |
| *Bacillus albus* | N35-10-2 | GCF_001884185.1 |
| *Bacillus anthracis* | Ames | GCF_000007845.1 |
| *Bacillus cereus sensu stricto* | ATCC 14579 | GCF_000007825.1 |
| *Bacillus cytotoxicus* | NVH 391-98 | GCF_000017425.1 |
| *Bacillus luti* | TD41 | GCF_001884105.1 |
| *Bacillus mobilis* | 0711P9-1 | GCF_001884045.1 |
| *Bacillus mycoides* | DSM 2048 | GCF_000003925.1 |
| *Bacillus nitratireducens* | 4049 | GCF_001884135.1 |
| *Bacillus pacificus* | EB422 | GCF_001884025.1 |
| *Bacillus paramycoides* | NH24A2 | GCF_001884235.1 |
| *Bacillus paranthracis* | MN5 | GCF_001883995.1 |
| *Bacillus proteolyticus* | TD42 | GCF_001884065.1 |
| *Bacillus pseudomycoides* | DSM 12442 | GCF_000161455.1 |
| *Bacillus thuringiensis* | Serovar Berliner ATCC 10792 | GCF_000161615.1 |
| *Bacillus toyonensis* | BCT-7112 | GCF_000496285.1 |
| *Bacillus tropicus* | N24 | GCF_001884035.1 |
| *Bacillus weihenstephanensis* | WSBC 10204 | GCF_000775975.1 |
| *Bacillus wiedmannii* | FSL W8-0169 | GCF_001583695.1 |

^a^For each *B. cereus* group species except *B. anthracis*, a type strain genome was selected to represent the species; for *B. anthracis*, the closed ResSeq reference genome was used in lieu of the scaffolded type strain genome
