# Supplementary material for: Characterization of Emetic and Diarrheal Bacillus cereus Strains From a 2016 Foodborne Outbreak Using Whole-Genome Sequencing: Addressing the Microbiological, Epidemiological, and Bioinformatic Challenges
Source: Front Microbiol. 2019 Feb 12;10:144. doi: 10.3389/fmicb.2019.00144 (PMC6379260; doi:10.3389/fmicb.2019.00144)
Supplement: Supplementary file 4 [file Table_4.DOCX]

**Supplementary Table S4.** All pairwise tests of tree topologies using a *Z* test based on the Kendall-Colijn metric^a^

| **Reference^b^** | **Query^b^** | **Z^c^** | ***P*-Value** | **Corrected *P*-Value^d^** |
| --- | --- | --- | --- | --- |
| AH187_CFSAN_NOdust_all | AH187_CFSAN_NOdust_core | -4.38 | 0 | 0 |
| AH187_CFSAN_NOdust_all | AH187_CFSAN_YESdust_all | -2.86 | 0.0021 | 0.3591 |
| AH187_CFSAN_NOdust_all | AH187_Freebayes_NOdust_all | -2.79 | 0.0027 | 0.4617 |
| AH187_CFSAN_NOdust_all | AH187_Freebayes_NOdust_core | -2.95 | 0.0016 | 0.2736 |
| AH187_CFSAN_NOdust_all | AH187_Freebayes_YESdust_all | -2.65 | 0.004 | 0.684 |
| AH187_CFSAN_NOdust_all | AH187_Freebayes_YESdust_core | -2.82 | 0.0024 | 0.4104 |
| AH187_CFSAN_NOdust_all | AH187_LYVE-SET_NOdust_all | -4.36 | 0 | 0 |
| AH187_CFSAN_NOdust_all | AH187_LYVE-SET_NOdust_core | -3.71 | 1.00E-04 | 0.0171 |
| AH187_CFSAN_NOdust_all | AH187_LYVE-SET_YESdust_all | -4.39 | 0 | 0 |
| AH187_CFSAN_NOdust_all | AH187_LYVE-SET_YESdust_core | -3.69 | 1.00E-04 | 0.0171 |
| AH187_CFSAN_NOdust_all | AH187_Parsnp_NOdust_core | -2.84 | 0.0022 | 0.3762 |
| AH187_CFSAN_NOdust_all | AH187_Parsnp_YESdust_core | -2.66 | 0.0039 | 0.6669 |
| AH187_CFSAN_NOdust_all | AH187_Samtools_NOdust_all | -2.81 | 0.0025 | 0.4275 |
| AH187_CFSAN_NOdust_all | AH187_Samtools_NOdust_core | -2.77 | 0.0028 | 0.4788 |
| AH187_CFSAN_NOdust_all | AH187_Samtools_YESdust_all | -2.82 | 0.0024 | 0.4104 |
| AH187_CFSAN_NOdust_all | AH187_Samtools_YESdust_core | -2.79 | 0.0026 | 0.4446 |
| AH187_CFSAN_NOdust_all | NOREF_kSNP_NAdust_all | -2.86 | 0.0021 | 0.3591 |
| AH187_CFSAN_NOdust_all | NOREF_kSNP_NAdust_core | -2.6 | 0.0047 | 0.8037 |
| AH187_CFSAN_NOdust_core | AH187_CFSAN_YESdust_all | -2.86 | 0.0021 | 0.3591 |
| AH187_CFSAN_NOdust_core | AH187_Freebayes_NOdust_all | -2.78 | 0.0027 | 0.4617 |
| AH187_CFSAN_NOdust_core | AH187_Freebayes_NOdust_core | -2.96 | 0.0015 | 0.2565 |
| AH187_CFSAN_NOdust_core | AH187_Freebayes_YESdust_all | -2.67 | 0.0038 | 0.6498 |
| AH187_CFSAN_NOdust_core | AH187_Freebayes_YESdust_core | -2.82 | 0.0024 | 0.4104 |
| AH187_CFSAN_NOdust_core | AH187_LYVE-SET_NOdust_all | -4.38 | 0 | 0 |
| AH187_CFSAN_NOdust_core | AH187_LYVE-SET_NOdust_core | -3.69 | 1.00E-04 | 0.0171 |
| AH187_CFSAN_NOdust_core | AH187_LYVE-SET_YESdust_all | -4.38 | 0 | 0 |
| AH187_CFSAN_NOdust_core | AH187_LYVE-SET_YESdust_core | -3.71 | 1.00E-04 | 0.0171 |
| AH187_CFSAN_NOdust_core | AH187_Parsnp_NOdust_core | -2.84 | 0.0022 | 0.3762 |
| AH187_CFSAN_NOdust_core | AH187_Parsnp_YESdust_core | -2.66 | 0.0039 | 0.6669 |
| AH187_CFSAN_NOdust_core | AH187_Samtools_NOdust_all | -2.82 | 0.0024 | 0.4104 |
| AH187_CFSAN_NOdust_core | AH187_Samtools_NOdust_core | -2.78 | 0.0027 | 0.4617 |
| AH187_CFSAN_NOdust_core | AH187_Samtools_YESdust_all | -2.81 | 0.0025 | 0.4275 |
| AH187_CFSAN_NOdust_core | AH187_Samtools_YESdust_core | -2.79 | 0.0027 | 0.4617 |
| AH187_CFSAN_NOdust_core | NOREF_kSNP_NAdust_all | -2.86 | 0.0022 | 0.3762 |
| AH187_CFSAN_NOdust_core | NOREF_kSNP_NAdust_core | -2.61 | 0.0046 | 0.7866 |
| AH187_CFSAN_YESdust_all | AH187_Freebayes_NOdust_all | -3.06 | 0.0011 | 0.1881 |
| AH187_CFSAN_YESdust_all | AH187_Freebayes_NOdust_core | -3.31 | 5.00E-04 | 0.0855 |
| AH187_CFSAN_YESdust_all | AH187_Freebayes_YESdust_all | -3.2 | 7.00E-04 | 0.1197 |
| AH187_CFSAN_YESdust_all | AH187_Freebayes_YESdust_core | -3.32 | 5.00E-04 | 0.0855 |
| AH187_CFSAN_YESdust_all | AH187_LYVE-SET_NOdust_all | -2.96 | 0.0015 | 0.2565 |
| AH187_CFSAN_YESdust_all | AH187_LYVE-SET_NOdust_core | -2.91 | 0.0018 | 0.3078 |
| AH187_CFSAN_YESdust_all | AH187_LYVE-SET_YESdust_all | -2.95 | 0.0016 | 0.2736 |
| AH187_CFSAN_YESdust_all | AH187_LYVE-SET_YESdust_core | -2.9 | 0.0019 | 0.3249 |
| AH187_CFSAN_YESdust_all | AH187_Parsnp_NOdust_core | -2.85 | 0.0022 | 0.3762 |
| AH187_CFSAN_YESdust_all | AH187_Parsnp_YESdust_core | -2.75 | 0.003 | 0.513 |
| AH187_CFSAN_YESdust_all | AH187_Samtools_NOdust_all | -3.27 | 5.00E-04 | 0.0855 |
| AH187_CFSAN_YESdust_all | AH187_Samtools_NOdust_core | -3.03 | 0.0012 | 0.2052 |
| AH187_CFSAN_YESdust_all | AH187_Samtools_YESdust_all | -3.11 | 9.00E-04 | 0.1539 |
| AH187_CFSAN_YESdust_all | AH187_Samtools_YESdust_core | -3.23 | 6.00E-04 | 0.1026 |
| AH187_CFSAN_YESdust_all | NOREF_kSNP_NAdust_all | -2.98 | 0.0014 | 0.2394 |
| AH187_CFSAN_YESdust_all | NOREF_kSNP_NAdust_core | -2.79 | 0.0027 | 0.4617 |
| AH187_Freebayes_NOdust_all | AH187_Freebayes_NOdust_core | -3.11 | 9.00E-04 | 0.1539 |
| AH187_Freebayes_NOdust_all | AH187_Freebayes_YESdust_all | -3.29 | 5.00E-04 | 0.0855 |
| AH187_Freebayes_NOdust_all | AH187_Freebayes_YESdust_core | -3.12 | 9.00E-04 | 0.1539 |
| AH187_Freebayes_NOdust_all | AH187_LYVE-SET_NOdust_all | -2.87 | 0.0021 | 0.3591 |
| AH187_Freebayes_NOdust_all | AH187_LYVE-SET_NOdust_core | -2.62 | 0.0045 | 0.7695 |
| AH187_Freebayes_NOdust_all | AH187_LYVE-SET_YESdust_all | -2.87 | 0.0021 | 0.3591 |
| AH187_Freebayes_NOdust_all | AH187_LYVE-SET_YESdust_core | -2.6 | 0.0046 | 0.7866 |
| AH187_Freebayes_NOdust_all | AH187_Parsnp_NOdust_core | -2.93 | 0.0017 | 0.2907 |
| AH187_Freebayes_NOdust_all | AH187_Parsnp_YESdust_core | -2.8 | 0.0026 | 0.4446 |
| AH187_Freebayes_NOdust_all | AH187_Samtools_NOdust_all | -3.12 | 9.00E-04 | 0.1539 |
| AH187_Freebayes_NOdust_all | AH187_Samtools_NOdust_core | -2.94 | 0.0017 | 0.2907 |
| AH187_Freebayes_NOdust_all | AH187_Samtools_YESdust_all | -3.27 | 5.00E-04 | 0.0855 |
| AH187_Freebayes_NOdust_all | AH187_Samtools_YESdust_core | -3.09 | 0.001 | 0.171 |
| AH187_Freebayes_NOdust_all | NOREF_kSNP_NAdust_all | -3.06 | 0.0011 | 0.1881 |
| AH187_Freebayes_NOdust_all | NOREF_kSNP_NAdust_core | -2.73 | 0.0031 | 0.5301 |
| AH187_Freebayes_NOdust_core | AH187_Freebayes_YESdust_all | -3.3 | 5.00E-04 | 0.0855 |
| AH187_Freebayes_NOdust_core | AH187_Freebayes_YESdust_core | -3.48 | 2.00E-04 | 0.0342 |
| AH187_Freebayes_NOdust_core | AH187_LYVE-SET_NOdust_all | -3.12 | 9.00E-04 | 0.1539 |
| AH187_Freebayes_NOdust_core | AH187_LYVE-SET_NOdust_core | -3.01 | 0.0013 | 0.2223 |
| AH187_Freebayes_NOdust_core | AH187_LYVE-SET_YESdust_all | -3.11 | 9.00E-04 | 0.1539 |
| AH187_Freebayes_NOdust_core | AH187_LYVE-SET_YESdust_core | -3.01 | 0.0013 | 0.2223 |
| AH187_Freebayes_NOdust_core | AH187_Parsnp_NOdust_core | -2.96 | 0.0015 | 0.2565 |
| AH187_Freebayes_NOdust_core | AH187_Parsnp_YESdust_core | -2.83 | 0.0023 | 0.3933 |
| AH187_Freebayes_NOdust_core | AH187_Samtools_NOdust_all | -3.43 | 3.00E-04 | 0.0513 |
| AH187_Freebayes_NOdust_core | AH187_Samtools_NOdust_core | -3.11 | 9.00E-04 | 0.1539 |
| AH187_Freebayes_NOdust_core | AH187_Samtools_YESdust_all | -3.3 | 5.00E-04 | 0.0855 |
| AH187_Freebayes_NOdust_core | AH187_Samtools_YESdust_core | -3.24 | 6.00E-04 | 0.1026 |
| AH187_Freebayes_NOdust_core | NOREF_kSNP_NAdust_all | -3.1 | 0.001 | 0.171 |
| AH187_Freebayes_NOdust_core | NOREF_kSNP_NAdust_core | -2.89 | 0.0019 | 0.3249 |
| AH187_Freebayes_YESdust_all | AH187_Freebayes_YESdust_core | -3.22 | 7.00E-04 | 0.1197 |
| AH187_Freebayes_YESdust_all | AH187_LYVE-SET_NOdust_all | -2.72 | 0.0032 | 0.5472 |
| AH187_Freebayes_YESdust_all | AH187_LYVE-SET_NOdust_core | -2.61 | 0.0045 | 0.7695 |
| AH187_Freebayes_YESdust_all | AH187_LYVE-SET_YESdust_all | -2.73 | 0.0032 | 0.5472 |
| AH187_Freebayes_YESdust_all | AH187_LYVE-SET_YESdust_core | -2.62 | 0.0044 | 0.7524 |
| AH187_Freebayes_YESdust_all | AH187_Parsnp_NOdust_core | -2.92 | 0.0017 | 0.2907 |
| AH187_Freebayes_YESdust_all | AH187_Parsnp_YESdust_core | -2.87 | 0.002 | 0.342 |
| AH187_Freebayes_YESdust_all | AH187_Samtools_NOdust_all | -3.09 | 0.001 | 0.171 |
| AH187_Freebayes_YESdust_all | AH187_Samtools_NOdust_core | -2.93 | 0.0017 | 0.2907 |
| AH187_Freebayes_YESdust_all | AH187_Samtools_YESdust_all | -3.01 | 0.0013 | 0.2223 |
| AH187_Freebayes_YESdust_all | AH187_Samtools_YESdust_core | -3.15 | 8.00E-04 | 0.1368 |
| AH187_Freebayes_YESdust_all | NOREF_kSNP_NAdust_all | -3.06 | 0.0011 | 0.1881 |
| AH187_Freebayes_YESdust_all | NOREF_kSNP_NAdust_core | -3.03 | 0.0012 | 0.2052 |
| AH187_Freebayes_YESdust_core | AH187_LYVE-SET_NOdust_all | -2.98 | 0.0015 | 0.2565 |
| AH187_Freebayes_YESdust_core | AH187_LYVE-SET_NOdust_core | -2.92 | 0.0018 | 0.3078 |
| AH187_Freebayes_YESdust_core | AH187_LYVE-SET_YESdust_all | -2.97 | 0.0015 | 0.2565 |
| AH187_Freebayes_YESdust_core | AH187_LYVE-SET_YESdust_core | -2.89 | 0.0019 | 0.3249 |
| AH187_Freebayes_YESdust_core | AH187_Parsnp_NOdust_core | -2.96 | 0.0015 | 0.2565 |
| AH187_Freebayes_YESdust_core | AH187_Parsnp_YESdust_core | -2.92 | 0.0018 | 0.3078 |
| AH187_Freebayes_YESdust_core | AH187_Samtools_NOdust_all | -3.41 | 3.00E-04 | 0.0513 |
| AH187_Freebayes_YESdust_core | AH187_Samtools_NOdust_core | -3.17 | 8.00E-04 | 0.1368 |
| AH187_Freebayes_YESdust_core | AH187_Samtools_YESdust_all | -3.31 | 5.00E-04 | 0.0855 |
| AH187_Freebayes_YESdust_core | AH187_Samtools_YESdust_core | -3.33 | 4.00E-04 | 0.0684 |
| AH187_Freebayes_YESdust_core | NOREF_kSNP_NAdust_all | -3.11 | 9.00E-04 | 0.1539 |
| AH187_Freebayes_YESdust_core | NOREF_kSNP_NAdust_core | -2.93 | 0.0017 | 0.2907 |
| AH187_LYVE-SET_NOdust_all | AH187_LYVE-SET_NOdust_core | -3.69 | 1.00E-04 | 0.0171 |
| AH187_LYVE-SET_NOdust_all | AH187_LYVE-SET_YESdust_all | -4.39 | 0 | 0 |
| AH187_LYVE-SET_NOdust_all | AH187_LYVE-SET_YESdust_core | -3.7 | 1.00E-04 | 0.0171 |
| AH187_LYVE-SET_NOdust_all | AH187_Parsnp_NOdust_core | -2.85 | 0.0022 | 0.3762 |
| AH187_LYVE-SET_NOdust_all | AH187_Parsnp_YESdust_core | -2.66 | 0.0039 | 0.6669 |
| AH187_LYVE-SET_NOdust_all | AH187_Samtools_NOdust_all | -2.8 | 0.0026 | 0.4446 |
| AH187_LYVE-SET_NOdust_all | AH187_Samtools_NOdust_core | -2.77 | 0.0028 | 0.4788 |
| AH187_LYVE-SET_NOdust_all | AH187_Samtools_YESdust_all | -2.82 | 0.0024 | 0.4104 |
| AH187_LYVE-SET_NOdust_all | AH187_Samtools_YESdust_core | -2.8 | 0.0026 | 0.4446 |
| AH187_LYVE-SET_NOdust_all | NOREF_kSNP_NAdust_all | -2.88 | 0.002 | 0.342 |
| AH187_LYVE-SET_NOdust_all | NOREF_kSNP_NAdust_core | -2.61 | 0.0045 | 0.7695 |
| AH187_LYVE-SET_NOdust_core | AH187_LYVE-SET_YESdust_all | -3.4 | 3.00E-04 | 0.0513 |
| AH187_LYVE-SET_NOdust_core | AH187_LYVE-SET_YESdust_core | -4.78 | 0 | 0 |
| AH187_LYVE-SET_NOdust_core | AH187_Parsnp_NOdust_core | -2.3 | 0.0107 | 1 |
| AH187_LYVE-SET_NOdust_core | AH187_Parsnp_YESdust_core | -2.19 | 0.0143 | 1 |
| AH187_LYVE-SET_NOdust_core | AH187_Samtools_NOdust_all | -2.4 | 0.0082 | 1 |
| AH187_LYVE-SET_NOdust_core | AH187_Samtools_NOdust_core | -2.33 | 0.01 | 1 |
| AH187_LYVE-SET_NOdust_core | AH187_Samtools_YESdust_all | -2.39 | 0.0083 | 1 |
| AH187_LYVE-SET_NOdust_core | AH187_Samtools_YESdust_core | -2.47 | 0.0067 | 1 |
| AH187_LYVE-SET_NOdust_core | NOREF_kSNP_NAdust_all | -2.41 | 0.0081 | 1 |
| AH187_LYVE-SET_NOdust_core | NOREF_kSNP_NAdust_core | -2.49 | 0.0063 | 1 |
| AH187_LYVE-SET_YESdust_all | AH187_LYVE-SET_YESdust_core | -3.69 | 1.00E-04 | 0.0171 |
| AH187_LYVE-SET_YESdust_all | AH187_Parsnp_NOdust_core | -2.85 | 0.0022 | 0.3762 |
| AH187_LYVE-SET_YESdust_all | AH187_Parsnp_YESdust_core | -2.66 | 0.0039 | 0.6669 |
| AH187_LYVE-SET_YESdust_all | AH187_Samtools_NOdust_all | -2.81 | 0.0025 | 0.4275 |
| AH187_LYVE-SET_YESdust_all | AH187_Samtools_NOdust_core | -2.79 | 0.0027 | 0.4617 |
| AH187_LYVE-SET_YESdust_all | AH187_Samtools_YESdust_all | -2.81 | 0.0024 | 0.4104 |
| AH187_LYVE-SET_YESdust_all | AH187_Samtools_YESdust_core | -2.8 | 0.0025 | 0.4275 |
| AH187_LYVE-SET_YESdust_all | NOREF_kSNP_NAdust_all | -2.86 | 0.0021 | 0.3591 |
| AH187_LYVE-SET_YESdust_all | NOREF_kSNP_NAdust_core | -2.62 | 0.0044 | 0.7524 |
| AH187_LYVE-SET_YESdust_core | AH187_Parsnp_NOdust_core | -2.29 | 0.0109 | 1 |
| AH187_LYVE-SET_YESdust_core | AH187_Parsnp_YESdust_core | -2.18 | 0.0147 | 1 |
| AH187_LYVE-SET_YESdust_core | AH187_Samtools_NOdust_all | -2.4 | 0.0083 | 1 |
| AH187_LYVE-SET_YESdust_core | AH187_Samtools_NOdust_core | -2.32 | 0.0101 | 1 |
| AH187_LYVE-SET_YESdust_core | AH187_Samtools_YESdust_all | -2.41 | 0.0081 | 1 |
| AH187_LYVE-SET_YESdust_core | AH187_Samtools_YESdust_core | -2.46 | 0.007 | 1 |
| AH187_LYVE-SET_YESdust_core | NOREF_kSNP_NAdust_all | -2.4 | 0.0082 | 1 |
| AH187_LYVE-SET_YESdust_core | NOREF_kSNP_NAdust_core | -2.49 | 0.0063 | 1 |
| AH187_Parsnp_NOdust_core | AH187_Parsnp_YESdust_core | -3.79 | 1.00E-04 | 0.0171 |
| AH187_Parsnp_NOdust_core | AH187_Samtools_NOdust_all | -2.8 | 0.0026 | 0.4446 |
| AH187_Parsnp_NOdust_core | AH187_Samtools_NOdust_core | -2.75 | 0.003 | 0.513 |
| AH187_Parsnp_NOdust_core | AH187_Samtools_YESdust_all | -2.82 | 0.0024 | 0.4104 |
| AH187_Parsnp_NOdust_core | AH187_Samtools_YESdust_core | -2.75 | 0.003 | 0.513 |
| AH187_Parsnp_NOdust_core | NOREF_kSNP_NAdust_all | -3.38 | 4.00E-04 | 0.0684 |
| AH187_Parsnp_NOdust_core | NOREF_kSNP_NAdust_core | -2.77 | 0.0028 | 0.4788 |
| AH187_Parsnp_YESdust_core | AH187_Samtools_NOdust_all | -2.6 | 0.0047 | 0.8037 |
| AH187_Parsnp_YESdust_core | AH187_Samtools_NOdust_core | -2.49 | 0.0063 | 1 |
| AH187_Parsnp_YESdust_core | AH187_Samtools_YESdust_all | -2.5 | 0.0062 | 1 |
| AH187_Parsnp_YESdust_core | AH187_Samtools_YESdust_core | -2.61 | 0.0046 | 0.7866 |
| AH187_Parsnp_YESdust_core | NOREF_kSNP_NAdust_all | -3.16 | 8.00E-04 | 0.1368 |
| AH187_Parsnp_YESdust_core | NOREF_kSNP_NAdust_core | -2.93 | 0.0017 | 0.2907 |
| AH187_Samtools_NOdust_all | AH187_Samtools_NOdust_core | -3.19 | 7.00E-04 | 0.1197 |
| AH187_Samtools_NOdust_all | AH187_Samtools_YESdust_all | -3.29 | 5.00E-04 | 0.0855 |
| AH187_Samtools_NOdust_all | AH187_Samtools_YESdust_core | -3.43 | 3.00E-04 | 0.0513 |
| AH187_Samtools_NOdust_all | NOREF_kSNP_NAdust_all | -3.08 | 0.001 | 0.171 |
| AH187_Samtools_NOdust_all | NOREF_kSNP_NAdust_core | -2.87 | 0.002 | 0.342 |
| AH187_Samtools_NOdust_core | AH187_Samtools_YESdust_all | -2.98 | 0.0014 | 0.2394 |
| AH187_Samtools_NOdust_core | AH187_Samtools_YESdust_core | -3.25 | 6.00E-04 | 0.1026 |
| AH187_Samtools_NOdust_core | NOREF_kSNP_NAdust_all | -2.87 | 0.002 | 0.342 |
| AH187_Samtools_NOdust_core | NOREF_kSNP_NAdust_core | -2.46 | 0.0069 | 1 |
| AH187_Samtools_YESdust_all | AH187_Samtools_YESdust_core | -3.23 | 6.00E-04 | 0.1026 |
| AH187_Samtools_YESdust_all | NOREF_kSNP_NAdust_all | -3.05 | 0.0011 | 0.1881 |
| AH187_Samtools_YESdust_all | NOREF_kSNP_NAdust_core | -2.73 | 0.0032 | 0.5472 |
| AH187_Samtools_YESdust_core | NOREF_kSNP_NAdust_all | -2.98 | 0.0014 | 0.2394 |
| AH187_Samtools_YESdust_core | NOREF_kSNP_NAdust_core | -2.74 | 0.0031 | 0.5301 |
| NA_kSNP_NAdust_all | NOREF_kSNP_NAdust_core | -2.96 | 0.0016 | 0.2736 |

^a^See Katz et al., 2017; Kendall and Colijn, 2015

^b^Names of reference and query phylogenies denote reference genome (“AH187” for reference-based pipelines, “NOREF” for reference-free kSNP pipeline), pipeline (“CFSAN”, “Freebayes”, “kSNP”, “LYVE-SET”, “Parsnp”, or “Samtools”), reference genome masking (“NOdust” for an unmasked reference genome, “YESdust” for a dustmasked reference genome, or “NAdust” for reference-free kSNP pipeline, for which dustmasking is not applicable), and SNPs used to construct the phylogeny (“core” for core SNPs, or “all” for core and accessory SNPs), separated by an underscore (“_”)

^c^Kendall and Colijn Z test statistic

^d^Corrected using a Bonferroni correction
