# Supplementary material for: Characterization of Emetic and Diarrheal Bacillus cereus Strains From a 2016 Foodborne Outbreak Using Whole-Genome Sequencing: Addressing the Microbiological, Epidemiological, and Bioinformatic Challenges
Source: Front Microbiol. 2019 Feb 12;10:144. doi: 10.3389/fmicb.2019.00144 (PMC6379260; doi:10.3389/fmicb.2019.00144)
Supplement: Supplementary file 5 [file Table_5.DOCX]

**Supplementary Table S5.** Annotated core SNPs identified in 30 clade III emetic isolates from a foodborne outbreak.^a^

| **SNP Position^b^** | **Feature Type** | **Feature Annotation** | **Feature Start** | **Feature End** | **Feature Strand** | **Protein Accession** | **AA Sub.^c^** | **Support^d^** |
| --- | --- | --- | --- | --- | --- | --- | --- | --- |
| 112,075 | NA | NA | NA | NA | NA | NA | NA | 5/5 (CFLPS) |
| 379,005 | Protein coding | hypothetical protein | 378,189 | 379,082 | + | WP_000375183.1 | V273M | 5/5 (CFLPS) |
| 1,598,949 | Protein coding | carboxypeptidase M32 | 1,598,521 | 1,600,038 | + | WP_000215096.1 | F143L | 5/5 (CFLPS) |
| 2,121,387 | Protein coding | GTP 3',8-cyclase MoaA | 2,121,122 | 2,122,135 | + | WP_000544623.1 | R89M | 5/5 (CFLPS) |
| 2,636,155 | Protein coding | chitin-binding protein | 2,634,899 | 2,636,266 | - | WP_000795732.1 | A38P | 5/5 (CFLPS) |
| 2,939,657 | Protein coding | D-alanyl-D-alanine carboxypeptidase | 2,939,210 | 2,940,337 | - | WP_000857793.1 | N227K | 5/5 (CFLPS) |
| 3,001,665 | Protein coding | ABC transporter substrate-binding protein | 3,000,787 | 3,003,339 | - | WP_000925107.1 | Q559K | 5/5 (CFLPS) |
| 3,250,727 | Protein coding | iron-hydroxamate ABC transporter substrate-binding protein | 3,250,656 | 3,251,564 | - | WP_000728195.1 | P280A | 5/5 (CFLPS) |
| 3,910,134 | Protein coding | Cof-type HAD-IIB family hydrolase | 3,910,128 | 3,910,913 | - | WP_000593229.1 | None | 5/5 (CFLPS) |
| 3,968,777 | Protein coding | bifunctional glutamate N-acetyltransferase/amino-acid acetyltransferase ArgJ | 3,967,991 | 3,969,217 | - | WP_080001949.1 | None | 5/5 (CFLPS) |
| 3,997,566 | Protein coding | sigma-54-dependent Fis family transcriptional regulator | 3,997,365 | 3,999,437 | - | WP_000811010.1 | None | 5/5 (CFLPS) |
| 4,004,536 | Protein coding | DNA repair protein RecN | 4,003,535 | 4,005,274 | - | WP_000947749.1 | N247D | 5/5 (CFLPS) |
| 4,340,331 | Protein coding | DNA polymerase/3'-5' exonuclease PolX | 4,339,214 | 4,340,932 | - | WP_000867556.1 | A201E | 5/5 (CFLPS) |
| 4,456,968 | NA | NA | NA | NA | NA | NA | NA | 5/5 (CFLPS) |
| 4,971,357 | Protein coding | isopeptide-forming domain-containing fimbrial protein | 4,970,610 | 4,973,537 | - | WP_001028699.1 | None | 5/5 (CFLPS) |
| 5,268,113 | Protein coding | OxaA precursor | 5,267,494 | 5,268,261 | - | WP_000727745.1 | Y50S | 5/5 (CFLPS) |
| 541,261 | NA | NA | NA | NA | NA | NA | NA | 4/5 (CF-PS) |
| 2,753,013 | Protein coding | VOC family protein | 2,752,892 | 2,753,281 | - | WP_000800664.1 | N90I | 4/5 (CF-PS) |
| 3,125,514 | Protein coding | histidine--tRNA ligase | 3,124,237 | 3,125,514 | + | WP_000425984.1 | *426Y | 4/5 (CF-PS) |
| 4,652,166 | tRNA | tRNA-Arg | 4652144 | 4652217 | - | NA | NA | 4/5 (CFL-S) |
| 4,795,148 | NA | NA | NA | NA | NA | NA | NA | 4/5 (CFL-S) |
| 1,554,370 | Protein coding | heptaprenyl diphosphate synthase component II | 1,553,963 | 1,554,925 | + | WP_000776087.1 | None | 4/5 (C-LPS) |
| 6,217 | Protein coding | DNA gyrase subunit B | 4,586 | 6,508 | + | WP_000435993.1 | Q544H | 4/5 (-FLPS) |
| 1,927,356 | NA | NA | NA | NA | NA | NA | NA | 3/5 (-F-PS) |
| 3,127,731 | Protein coding | NAD(P)H-dependent oxidoreductase | 3,127,411 | 3,128,094 | + | WP_000185741.1 | M107I | 3/5 (-F-PS) |
| 4,855,496 | Protein coding | AraC family transcriptional regulator | 4,855,079 | 4,855,924 | - | WP_000383877.1 | None | 3/5 (C-LP-) |
| 484,550 | Protein coding | cell wall anchor protein | 483,608 | 484,786 | - | WP_000718025.1 | None | 2/5 (-F--S) |
| 947,445 | NA | NA | NA | NA | NA | NA | NA | 2/5 (-F--S) |
| 2,378,322 | Protein coding | collagen-like repeat preface domain-containing protein | 2,377,819 | 2,379,084 | + | WP_000019171.1 | None | 2/5 (-F--S) |
| 2,397,774 | NA | NA | NA | NA | NA | NA | NA | 2/5 (-F--S) |
| 4,471,388 | Protein coding | DNA translocase FtsK | 4,468,892 | 4,472,704 | - | WP_000888073.1 | None | 2/5 (-F--S) |
| 280,778 | NA | NA | NA | NA | NA | NA | NA | 2/5 (-FL--) |

^a^NA, not applicable; SNP was identified in an unannotated region

^b^Position in *B. cereus* str. AH187 chromosome

^c^AA Sub., amino acid substitution; synonymous SNP changes are denoted by “None”, and stop codons are denoted by *

^d^The number of reference-based SNP calling pipelines in which a SNP at a given position was identified is denoted by n/5, where 2 ≤ n ≤ 5; the pipelines that identified a given SNP are in parentheses, with the letters C, F, L, P, and S representing the CFSAN, Freebayes, LYVE-SET, Parsnp, and Samtools pipelines, respectively
