# Supplementary material for: Characterization of Emetic and Diarrheal Bacillus cereus Strains From a 2016 Foodborne Outbreak Using Whole-Genome Sequencing: Addressing the Microbiological, Epidemiological, and Bioinformatic Challenges
Source: Front Microbiol. 2019 Feb 12;10:144. doi: 10.3389/fmicb.2019.00144 (PMC6379260; doi:10.3389/fmicb.2019.00144)
Supplement: Supplementary file 6 [file Data_Sheet_1.PDF]

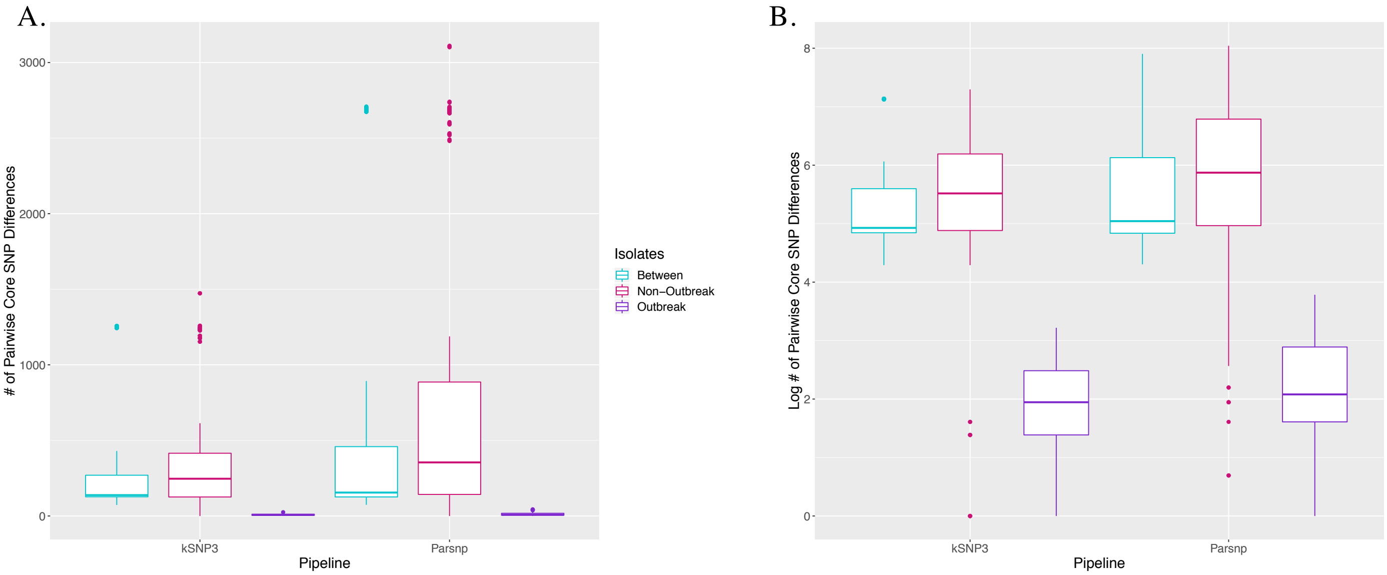

Supplementary Figure S1. Ranges of pairwise (A) core SNP differences and (B) log-transformed core SNP differences (i) among 30 emetic group III ST 26 *B. cereus* group strains isolated in conjunction with the foodborne outbreak described here ("Outbreak", in purple), (ii) among all other emetic group III ST 26 genomes available in NCBI (n = 25; "Non-Outbreak", shown in magenta), and (iii) between the 30 emetic group III ST 26 *B. cereus* group strains isolated here and the 25 emetic group III ST 26 genomes available in NCBI ("Between", shown in teal). Two SNP calling pipelines that use assembled genomes as input (reference-based pipeline Parsnp, which was used with the raw/unmasked chromosome of emetic group III ST 26 *B. cereus* str. AH187, and reference-free kSNP3) were tested. Lower and upper box hinges correspond to the first and third quartiles, respectively. Lower and upper whiskers extend from the hinge to the smallest and largest values no more distant than 1.5 times the interquartile range from the hinge, respectively. Points represent pairwise distances that fall beyond the ends of the whiskers.
